# Phytochemical Profiling and Toxicological Evaluation of *Atraphaxis virgata* and *Atraphaxis pyrifolia* Extracts Using GC–MS and LC–MS

**DOI:** 10.3390/molecules31111795

**Published:** 2026-05-23

**Authors:** Meruyert D. Dauletova, Almagul K. Umbetova, Aisulu Zh. Kabdraisova, Rizvangul S. Iminova, Gauhar Sh. Burasheva, Yuliya A. Litvinenko, Nazym S. Yelibayeva, Natalya V. Kurbatova, Dmitriy Yu. Korul’kin, Nailya A. Ibragimova, Gulnar O. Bugubaeva, Murat R. Zhumabayev

**Affiliations:** 1 Department of Chemistry and Technology of Organic Substances, Chemistry of Natural Compounds and Polymers, Faculty of Chemistry and Chemical Technology, Farabi University, Al-Farabi Ave. 71, Almaty 050040, Kazakhstan; dmd_09@inbox.ru (M.D.D.); gauharbur@mail.ru (G.S.B.); yuliya_litvinenk@mail.ru (Y.A.L.); nazym_yelibaeva@mail.ru (N.S.Y.); dmitriy.korulkin@kaznu.edu.kz (D.Y.K.); 2 Scientific Research Institute for New Chemical Technologies and Materials, Farabi University, 96a Tole Bi Str., Almaty 050012, Kazakhstan; zhaksais@gmail.com; 3 Branch Office of the Republican State Enterprise “Infracos”, Almaty 050046, Kazakhstan; 4 Department of Botany and Agrochemistry, Faculty of Biology and Biotechnology, Farabi University, Al-Farabi Ave. 71, Almaty 050040, Kazakhstan; kurbatova_nv77@mail.ru; 5 Scientific Center for Anti-Infectious Drugs, Laboratory of Pharmacology and Toxicology, Al-Farabi Ave. 75A, Almaty 050060, Kazakhstan; nailya.73@mail.ru; 6 Department of Chemistry, Chemical Technology and Ecology, Faculty of Biotechnology and Chemical Technologies, Almaty Technological University, Furkat Street 348/4, Almaty 050061, Kazakhstan; bugubaevagulnar@gmail.com; 7 Department of Chemistry, Institute of Natural Sciences, Kazakh National Women’s Teacher Training University, Gogol Str. 114 k1, Almaty 050000, Kazakhstan; alaman1977@gmail.com

**Keywords:** phytochemical profiling, GC–MS, LC–MS, flavonoids, phenolic compounds, *Atraphaxis*

## Abstract

*Atraphaxis virgata* and *Atraphaxis pyrifolia* are xerophytic species of the Polygonaceae family that remain insufficiently characterized from pharmacognostic, phytochemical, and toxicological perspectives. This study provides an integrated evaluation of both species through anatomical authentication, sequential extraction of CO_2_-extracted residual biomass, GC–MS and LC–MS metabolite profiling, and acute oral toxicity assessment. Anatomical analysis revealed shared xeromorphic traits, including cuticular protection, dorsiventral mesophyll organization, structured vascular bundles, and calcium oxalate druses. It also identified species-specific differences in leaf thickness, mesophyll arrangement, vascular architecture, and druse morphology. GC–MS analysis showed distinct chemical profiles: *A. virgata* displayed a concentrated profile dominated by acetophenone- and benzofuran-related constituents, whereas *A. pyrifolia* showed a broader spectrum of carbohydrate-derived, phenolic-related, and oxygenated constituents. LC–MS analysis supported the tentative annotation of diverse polyphenolic classes, including flavonoids, phenolic acids, coumarins, and phenylpropanoid derivatives. Acute oral toxicity testing showed no mortality at doses up to 2000 mg/kg, supporting a low acute oral toxicity classification under the tested conditions. However, histological examination revealed mild to moderate dose-dependent alterations in liver and kidney tissues at higher doses. The novelty of this work lies in linking diagnostic anatomical traits, species-specific metabolite patterns, residual biomass valorization, and preliminary safety evidence within a single comparative framework. These findings provide a basis for pharmacognostic authentication, phytochemical standardization, and future bioactivity-guided evaluation of *Atraphaxis* species.

## 1. Introduction

Kazakhstan’s flora comprises approximately 5850 plant species, including cultivated and introduced taxa. Most of these species are herbaceous, and more than 730 are endemic. Of the endemic species, 175 occur in steppe regions, 250 in desert and semi-desert zones, and approximately 600 are classified as rare or endangered. More than 1000 species are used in traditional medicine [1,2,3]. Recent advances in phytochemical profiling based on high-resolution chromatographic and spectrometric techniques have improved the detection and characterization of bioactive compounds in medicinal plants [4]. The genus *Atraphaxis* belongs to the family Polygonaceae and includes numerous species and subspecies [5,6,7,8]. Species of this genus commonly occur in arid and semi-arid habitats, including rocky slopes, foothill regions, and desert margins [5,6,7,8]. In Kazakhstan, *Atraphaxis* species are found on rocky and gravelly mountain slopes, often in saline, stony, and coarse substrates where shrub or semi-shrub communities develop [5,6]. In Turkmen traditional medicine, powdered shoots of *A. pyrifolia* are used to treat gastroenteritis [9]. Ethnobotanical reports from Uzbekistan also describe the use of *A. pyrifolia* leaf infusions to support cardiac function and circulation and to relieve headaches and insomnia [10].

*Atraphaxis* species have shown promising pharmacological properties, including antioxidant and antimicrobial activities [11]. Traditional uses for lymphatic disorders, bacterial fevers, throat infections, and ocular diseases such as cataracts have also been reported [11]. These effects may be related to the phytochemical composition of the genus. Earlier phytochemical studies showed that *Atraphaxis* species contain diverse compound classes, including phenylpropanoids, flavonoids, anthraquinones, sterols, benzoids, tannins, and other phenolic compounds [12]. Modern analytical approaches, particularly GC–MS and HPLC–MS, are now essential for the characterization of plant secondary metabolites because of their sensitivity and structural-informative capacity [13,14,15,16,17]. Flavonoid glycosides have been reported as major constituents of *Atraphaxis* species [18], and this compound class is widely associated with antioxidant, anti-inflammatory, and broader pharmacological activities [5,6,19,20,21,22,23]. Earlier phytochemical investigations of *A. pyrifolia* leaves identified 7-O-methylgossypetin-3-O-L-rhamnopyranoside [24], 7-O-methylluteolin 4′-O-β-D-glucofuranosyl-(1→6)-D-glucopyranoside [25], 8-acetylmethylgossypetin-3-O-L-rhamnopyranoside (pyrifolin), its aglycone 8-acetyl-7-methylgossypetin (pyrifolidin), and 7-methylgossypetin-3-O-L-rhamnopyranoside (pyrifolinin) [26]. Additional compounds, including 7-methylgossypetin 8-β-D-glucopyranoside 3-O-L-rhamnopyranoside and 7-methylgossypetin 8-β-D-glucopyranoside, have also been reported [27]. Recent GC–MS analysis of the hexane extract of *A. pyrifolia* leaves revealed γ-sitosterol, lup-20(29)-en-3-one, and nonacosane as major nonpolar constituents [5]. Detailed anatomical and morphological studies of *Atraphaxis* leaves and stems have contributed to accurate taxonomic classification [7]. Members of the genus are dwarf shrubs or shrubs, usually 30–150 cm in height, with elongated leaves and short axillary branchlets that are characteristic of the group [8].

Despite these reports, the novelty and regulatory relevance of the present study remain important. To date, no EFSA scientific opinion or regulatory safety assessment has been established for *Atraphaxis virgata* or *Atraphaxis pyrifolia*, highlighting the lack of toxicological and safety data for these species within the European regulatory framework. In addition, these species are not included in major international pharmacopoeias, including the European Pharmacopoeia, United States Pharmacopeia, British Pharmacopoeia, or Chinese Pharmacopoeia, and no official monographs defining their identity, quality control parameters, or safety profiles are currently available. Therefore, integrated data on anatomical authentication, phytochemical profiling, and in vivo toxicity are needed to support future standardization and safety evaluation.

The present study was designed to provide an integrated pharmacognostic, phytochemical, and toxicological evaluation of *A. virgata* and *A. pyrifolia*. Unlike previous studies that focused mainly on isolated phytochemical constituents or limited botanical descriptions, this work is the first direct comparative study of *A. virgata* and *A. pyrifolia* integrating morphometric anatomical analysis, investigation of CO_2_-extracted residual biomass, ultrasound-assisted ethanol extraction, GC–MS profiling, LC–MS/QTOF-based polyphenolic fingerprinting, and in vivo acute oral toxicity assessment under the same experimental conditions. This design allows botanical identity, extraction strategy, chemical composition, and preliminary safety to be evaluated together. Therefore, provides baseline data for authentication, standardization, and future pharmacological investigation of these under-studied xerophytic species.

## 2. Results and Discussion

The results are presented as an integrated comparative workflow linking anatomical authentication, extraction strategy, chemical profiling, and preliminary safety evaluation. This structure clarifies the descriptive characteristics of each species and their relevance for pharmacognostic standardization and future development of plant-derived bioactive preparations.

### 2.1. Anatomical and Morphological Study of the Leaves and Stems of Atraphaxis pyrifolia Bunge

**Figure 1:**
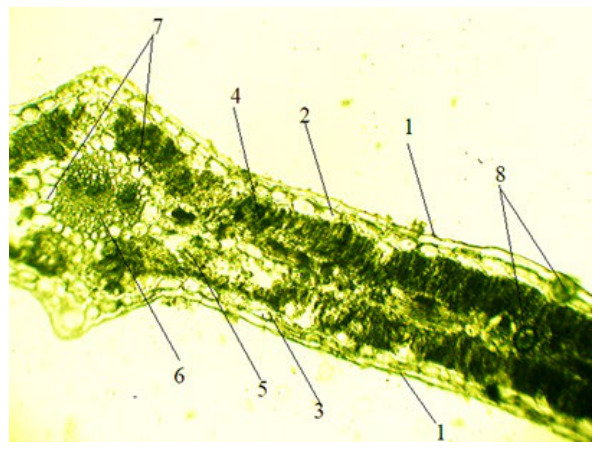
Anatomical structure of the leaf of *Atraphaxis pyrifolia* Bunge (×100): (1) cuticle; (2) upper epidermis; (3) lower epidermis; (4) palisade mesophyll; (5) spongy mesophyll; (6) vascular bundle; (7) bundle sheath cells; (8) druse crystals.

The anatomical and morphological structure of the leaf blade of *Atraphaxis pyrifolia* Bunge is shown in Figure 1.

The leaf blade of *A. pyrifolia* is covered by a cuticle on both adaxial and abaxial surfaces. Beneath the cuticle, the epidermis consists of relatively large rounded to rectangular cells. The lower epidermis (0.513 µm) is slightly thicker than the upper epidermis (0.482 µm). The leaf has a dorsiventral structure, with the mesophyll differentiated into palisade and spongy parenchyma. Sparse simple trichomes occur on the epidermal surface, and the epidermal layers are continuous at the rounded leaf margins. The biometric characteristics of five representative leaf samples are presented in Table 1. In these samples, mean total leaf thickness was 21.389 ± 1.456 µm, and mesophyll thickness was 15.831 ± 2.892 µm. The palisade and spongy mesophyll layers measured 7.476 ± 1.417 µm and 7.550 ± 1.483 µm, respectively, indicating nearly equal development of both layers. The palisade mesophyll consists of two layers of elongated cells beneath the upper epidermis, whereas the spongy mesophyll is loosely arranged toward the lower surface and contains large intercellular spaces.

The central vascular bundle, 245.06 µm in diameter, is located in the middle of the leaf blade and is accompanied by smaller lateral vascular bundles toward the margins. The vascular bundles are closed collateral bundles composed of phloem and xylem elements. Rounded bundle sheath cells surround the central bundle. Isolated sclerenchyma cells occur along the phloem, whereas the smaller vascular bundles lack sclerenchymatous cells. A diagnostic feature of *A. pyrifolia* is the presence of rounded calcium oxalate druses with an average diameter of 5.780 ± 1.747 µm. Their variable size reflects heterogeneity in mineral deposition and is consistent with diagnostic anatomical features reported for Polygonaceae [5].

**Figure 2:**
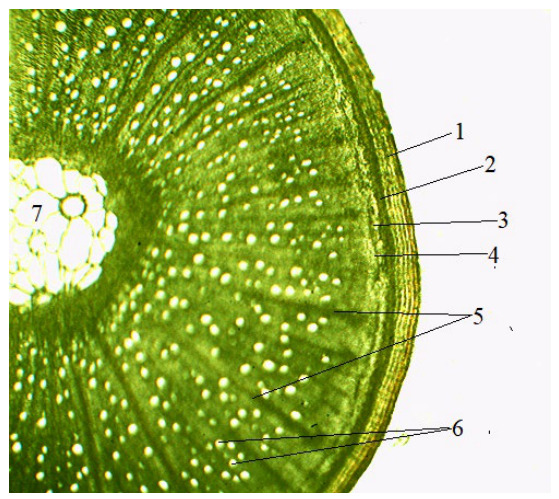
Anatomical structure of the stem of *Atraphaxis pyrifolia* (×70). 1—periderm; 2—collenchyma; 3—primary cortex parenchyma; 4—cambial zone; 5—medullary (vascular) rays; 6—xylem vessels; 7—pith parenchyma.

The anatomical and morphological structure of the stem of *Atraphaxis pyrifolia* Bunge is shown in Figure 2.

The stem surface is smooth to slightly ribbed and is covered by a periderm. The superficial periderm consists of 4–5 cell layers arranged in regular radial rows, of which 3–4 layers represent phelloderm. Mean periderm thickness was 1.090 µm. Beneath the periderm, a compact lamellar collenchyma layer is present, with a mean thickness of 0.553 µm. These tightly packed cells provide mechanical support within the primary cortex. Toward the central cylinder, the primary cortex parenchyma contains small inclusions in the form of calcium oxalate druses. The cambial zone consists of rectangular cells bordering the secondary tissues. Its mean thickness was 1.670 µm, while the primary cortex parenchyma measured 14.348 µm.

The central cylinder is located inward from the cortex. Lignified cells separated by parenchyma occur at the boundary between the cortex and the central cylinder. Numerous vascular rays formed by cambial activity are present. Xylem vessels are abundant and variable in size, with a mean diameter of 0.338 µm. The phloem is associated with sclerenchymatous fibers that form a supportive sheath. The central stem region consists of pith parenchyma composed of large, rounded, densely arranged cells. The peripheral region of the pith forms a perimedullary zone with smaller, thick-walled cells. The diameter of the central cylinder was 340.39 µm.

The biometric characteristics of *Atraphaxis pyrifolia* stems are presented in Table 2.

The biometric characteristics of five representative *Atraphaxis pyrifolia* stem samples are summarized in Table 2. The measured parameters showed low to moderate variability. Mean periderm thickness was 1.090 ± 0.252 µm, and the collenchyma layer measured 0.553 ± 0.156 µm, indicating consistent development of protective and mechanical tissues. The cambial zone had a mean thickness of 1.670 ± 0.183 µm, suggesting stable secondary growth activity. The primary cortex parenchyma measured 14.348 ± 1.462 µm and represented the largest part of the cortical region. The diameter of the central cylinder remained constant at 340.39 µm across all measurements, indicating structural uniformity of the conductive core. Xylem vessel diameter (0.338 ± 0.107 µm) showed moderate variability, reflecting differences in vessel size distribution within the vascular tissue.

### 2.2. Anatomical and Morphological Study of the Leaves and Stems of Atraphaxis virgata (Regel) Krasn

**Figure 3:**
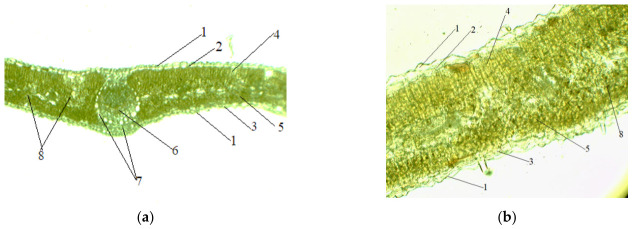
Anatomical structure of the leaf of *Atraphaxis virgata* (Regel) Krasn.: 1—cuticle; 2—upper epidermis; 3—lower epidermis; 4—palisade mesophyll; 5—spongy mesophyll with intercellular spaces; 6—central vascular bundle; 7—bundle sheath cells; 8—druse crystals. (**a**) Transverse section of the leaf blade (×70); (**b**) anatomical fragment of the leaf blade (×100).

The anatomical and morphological structure of the leaf blade of *Atraphaxis virgata* (Regel) Krasn. is shown in Figure 3.

The leaf blade of *A. virgata* is covered on both surfaces by a thick cuticular layer. Rounded upper epidermal cells (0.386 µm) and elongated-rounded lower epidermal cells (0.419 µm) are present beneath the cuticle. As in *A. pyrifolia*, the lower epidermal cells are slightly larger. The mesophyll is differentiated into palisade and spongy parenchyma, indicating a dorsiventral leaf structure. However, the total leaf thickness of *A. virgata* (11.758 µm) is almost two times lower than that of *A. pyrifolia*, which represents a diagnostic difference between the species. Sparse simple trichomes are present, and the epidermis forms a continuous layer on both leaf surfaces.

The palisade mesophyll is arranged in two layers beneath the upper epidermis. Unlike *A. pyrifolia*, the spongy mesophyll of *A. virgata* is more compact and contains fewer intercellular spaces. Total mesophyll thickness was 8.204 µm, with palisade and spongy layers measuring 5.002 µm and 3.732 µm, respectively. The difference between these layers (1.270 µm) is more pronounced than in *A. pyrifolia*, indicating stronger structural differentiation of the photosynthetic tissues. A central vascular bundle, 71.915 µm in diameter, is located in the midrib region. Compared with *A. pyrifolia*, *A. virgata* has a larger number of vascular bundles, although they are smaller in size. All vascular bundles are closed collateral and consist of xylem and phloem. The central bundle is surrounded by parenchymatous bundle sheath cells, and occasional sclerenchyma cells occur along the phloem. A diagnostic anatomical feature of *A. virgata* is the presence of numerous small calcium oxalate druses, with a mean diameter of 0.680 µm. In contrast, *A. pyrifolia* contains fewer but larger druses. This difference suggests species-specific variation in mineral deposition and stress-related physiological responses [28].

The biometric characteristics of five representative leaf samples of *A. virgata* are presented in Table 3.

**Figure 4:**
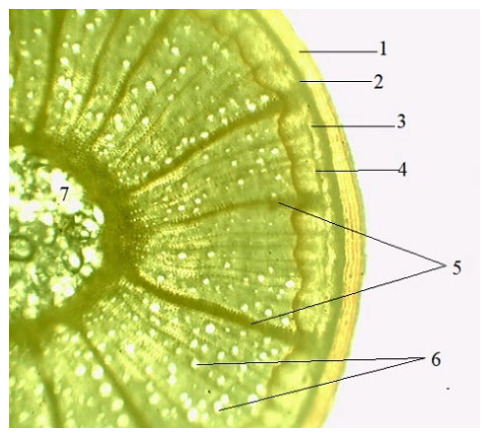
Anatomical structure of the stem of *Atraphaxis virgata* (×70): 1—periderm; 2—collenchyma; 3—primary cortex parenchyma; 4—cambial zone; 5—medullary (vascular) rays; 6—xylem vessels; 7—pith parenchyma.

The anatomical structure of the stem of *A. virgata* is shown in Figure 4.

The stem surface is smooth and covered by a periderm. The outer periderm consists of 4–5 layers of cells arranged in regular radial rows, similar to *A. pyrifolia*. The periderm thickness averages 1.084 µm. Beneath it lies a compact layer of lamellar collenchyma (0.476 µm), forming a mechanical support system within the primary cortex. The primary cortex parenchyma contains small calcium oxalate druse inclusions. The cambial zone, composed of rectangular cells, has an average thickness of 1.554 µm, while the total thickness of the primary cortex parenchyma is 13.310 µm. The central cylinder is well developed, with a diameter of 325.250 µm. At the boundary between the cortex and central cylinder, lignified cells separated by parenchyma are present. Numerous medullary rays formed by cambial activity are observed. Xylem vessels are less numerous than in *A. pyrifolia* but exhibit larger diameters (0.373 µm on average), indicating differences in hydraulic architecture. The phloem is associated with sclerenchymatous fibers forming a supportive sheath. The central part of the stem consists of pith parenchyma composed of large, rounded, densely packed cells. The peripheral pith region (perimedullary zone) contains smaller, thick-walled cells.

The biometric characteristics of five representative *A. virgata* stem samples are presented in Table 4.

Both *A. pyrifolia* and *A. virgata* share xeromorphic anatomical traits that reflect adaptation to arid and semi-arid environments. In both species, the leaves have a protective cuticular layer and dorsiventral organization with palisade and spongy mesophyll. The vascular system consists of closed collateral bundles, with the central bundle surrounded by parenchymatous bundle sheath cells. The stems are covered by periderm and contain compact collenchyma, a cambial zone, lignified elements at the cortex-cylinder boundary, xylem vessels, phloem-associated sclerenchymatous fibers, and central pith parenchyma. These shared traits are consistent with anatomical adaptations commonly reported in xerophytic shrubs, where protective tissues and vascular organization support water conservation and mechanical stability under drought conditions [29,30]. Despite these similarities, several diagnostically relevant differences were identified. *A. virgata* leaves were markedly thinner (11.758 µm) than *A. pyrifolia* leaves, indicating reduced structural investment in leaf tissue. The spongy mesophyll of *A. virgata* was more compact and contained fewer intercellular spaces, whereas that of *A. pyrifolia* was more loosely arranged with well-developed air spaces. The difference between palisade and spongy mesophyll thickness was also more pronounced in *A. virgata*, suggesting stronger functional differentiation of photosynthetic tissues. Vascular organization differed as well: *A. virgata* had more numerous but smaller vascular bundles, whereas *A. pyrifolia* had fewer but larger bundles. These differences may reflect distinct hydraulic strategies and resource allocation patterns [30].

The two species also differed in calcium oxalate druse formation. *A. pyrifolia* contained fewer but larger druses, while *A. virgata* contained numerous smaller crystals. This contrast may reflect species-specific patterns of mineral deposition and physiological responses to environmental stress, particularly in ion regulation and detoxification [30]. Stem anatomy further distinguished the two species. The stem of *A. virgata* was smooth, whereas that of *A. pyrifolia* was slightly ribbed. Xylem vessels in *A. virgata* were fewer but larger, suggesting a tendency toward higher hydraulic efficiency. In contrast, *A. pyrifolia* showed more numerous but smaller vessels, a feature that may enhance hydraulic safety under drought by reducing embolism risk [28,29].

Overall, both species share a xeromorphic structural framework, but differences in leaf thickness, mesophyll organization, vascular architecture, and druse formation indicate distinct adaptive strategies within the genus *Atraphaxis*. *A. virgata* appears to use a more conservative water-use strategy, characterized by thinner leaves and compact tissue organization. *A. pyrifolia* shows features associated with greater structural robustness and potentially enhanced hydraulic safety. From a pharmacognostic perspective, these anatomical differences provide diagnostic characters that can help distinguish raw materials of *A. virgata* and *A. pyrifolia*. Such differentiation is important for quality control, botanical authentication, and prevention of species substitution during future phytochemical or pharmaceutical development. Because the biometric analysis was based on five representative samples per organ and species, the measurements should be interpreted as descriptive anatomical data rather than population-level estimates.

### 2.3. Subcritical Carbon Dioxide Extraction Method

Subcritical CO_2_ extraction of the aerial parts of *A. virgata* and *A. pyrifolia* was performed at LLP “Pharmaceutical Manufacturer ZHANAFARM” using a UUPE-5L extraction unit. Liquid carbon dioxide [31] was used as the extractant. A total of 2600 g of plant material was processed. Before extraction, the raw material was milled to a particle size of 3–5 mm to increase the contact area between the plant matrix and the solvent.

Extraction was performed at a working pressure of 57–65 kgf/cm^2^ and a temperature of 18–23 °C for 8 h per loading cycle. The extraction parameters and yields are presented in Table 5. Under identical conditions, the CO_2_ extract yield was 5 g (7.46%) for *A. virgata* aerial parts and 13 g (9.3%) for *A. pyrifolia*.

The higher extract yield obtained from *A. pyrifolia* suggests species-dependent differences in CO_2_-soluble constituents. The residual CO_2_-extracted biomass was subsequently used for ultrasound-assisted hydroethanolic extraction to determine whether chemically valuable compounds remained after lipophilic extraction.

### 2.4. Ultrasound-Assisted Extraction of CO_2_-Extracted Residual Biomass (Meal)

The residual biomass (CO_2_-extracted meal) of *A. virgata* and *A. pyrifolia* obtained after subcritical CO_2_ extraction was subjected to ultrasound-assisted extraction. A 70% aqueous ethanol solution was used as the solvent at a raw material-to-solvent ratio of 1:8. Extraction was performed at 30–40 °C for 120 min under ultrasonic treatment in two consecutive stages. The hydroalcoholic extracts were filtered, combined, and concentrated to dryness under reduced pressure at 40–45 °C [32,33,34]. The sequential extraction strategy was based on solvent selectivity. Subcritical CO_2_ extraction primarily targets nonpolar and lipophilic constituents, including waxes, fatty-acid-related compounds, terpenoid-like metabolites, and other CO_2_-soluble components. In contrast, subsequent 70% hydroethanolic extraction of the residual biomass was intended to recover more polar and semi-polar constituents that remained in the plant matrix after lipophilic extraction, particularly phenolic acids, flavonoids, coumarins, phenylpropanoid derivatives, and carbohydrate-derived compounds. Therefore, the chemical profile of the hydroethanolic extract should be interpreted as the profile of the residual biomass fraction rather than the complete native phytochemical profile of the plant material. This sequence explains why chemically valuable polyphenolic constituents were still detected after CO_2_ extraction and supports more complete utilization of *Atraphaxis* biomass.

### 2.5. GC–MS Analysis of Ethanol Extracts from CO_2_-Extracted Biomass

The chemical composition of ethanol extracts obtained from the CO_2_-extracted biomass of *Atraphaxis virgata* and *Atraphaxis pyrifolia* was investigated using gas chromatography coupled with mass spectrometry (GC–MS). For analysis, 0.5 g of each extract was dissolved in 2 mL of hexane and injected into the chromatographic system. Data processing included determination of retention times and peak areas, followed by interpretation of mass spectra using the Wiley 7th Edition and NIST’02 spectral libraries, which contain more than 550,000 reference spectra. The GC–MS library-based tentative annotations and relative peak areas are presented in Table 6 and Table 7. Because authentic reference standards were not used, the assignments should be interpreted as tentative annotations rather than confirmed compound identifications, in accordance with current recommendations for mass spectrometry-based metabolite annotation and reporting [35]. Compounds with library match probabilities below 90% were interpreted cautiously and were not used as the sole basis for definitive biological conclusions.

The ethanol extract of *A. virgata* (Table 6) showed a dominant chromatographic peak with a relative peak area of 45.26%, tentatively assigned by library matching to an acetophenone-related compound. Because the library match probability was below 90% and authentic standards were not used, this peak is interpreted at the putative compound-class level rather than as a confirmed individual compound. The chromatographic profile therefore suggests the presence of acetophenone-related constituents, but confirmation using authentic standards or complementary structural analysis is required. The second major peak, with a relative peak area of 17.58%, was tentatively assigned as a benzofuran/coumaran-type constituent. Because its match probability was also below 90%, this assignment was treated as putative.

Among the phenolic-related annotations, 2-methoxyphenol showed a high library match probability (>90%), whereas vanillin and methyl vanillate were assigned with lower match probabilities and should therefore be considered putative. Consequently, biological interpretation was limited to the presence of phenolic-related compound classes rather than definitive activity attribution to individual low-confidence compounds [36]. The presence of n-hexadecanoic acid (1.82%) reflects common lipid-derived constituents; however, its biological role is context-dependent and should be interpreted cautiously. In contrast, the ethanol extract of *A. pyrifolia* (Table 7) showed a more complex chromatographic profile with several major peaks assigned by library matching to carbohydrate-derived and oxygenated compound classes. Because several assignments had library match probabilities below 90%, they should be interpreted as tentative or putative annotations rather than confirmed individual compounds. These findings suggest that the residual biomass retained a substantial proportion of hydrophilic metabolites after CO_2_ extraction.

Tentatively annotated bioactive-related compound classes in *A. pyrifolia* included phenolic, oxygenated, fatty-acid-related, and acetophenone-related constituents. Among these, the acetophenone-related annotation 2′,6′-dihydroxy-4′-methoxyacetophenone showed a high library match probability, whereas several other annotations had lower match probabilities and were therefore interpreted cautiously [36]. Linoleic acid (0.95%), an essential fatty acid, further contributes to the potential biological relevance of the extract due to its role in membrane function and inflammation modulation. Notably, acetophenone derivatives (8.28%) were also detected, indicating a shared chemotaxonomic feature between the two species, although with lower abundance compared to *A. virgata*.

**Figure 5:**
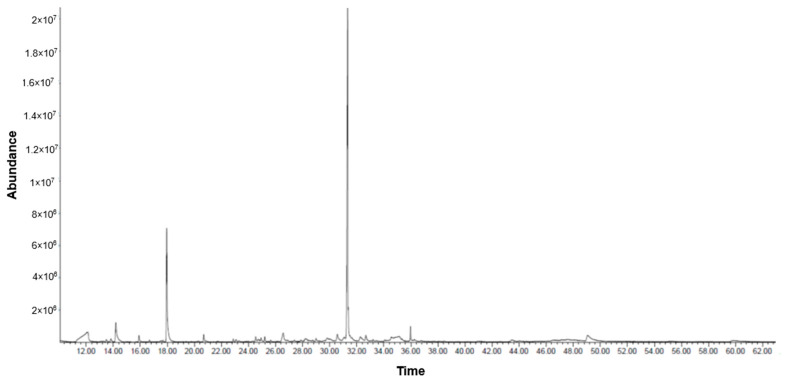
GC–MS chromatogram of the ethanol extract of *Atraphaxis virgata*.

**Figure 6:**
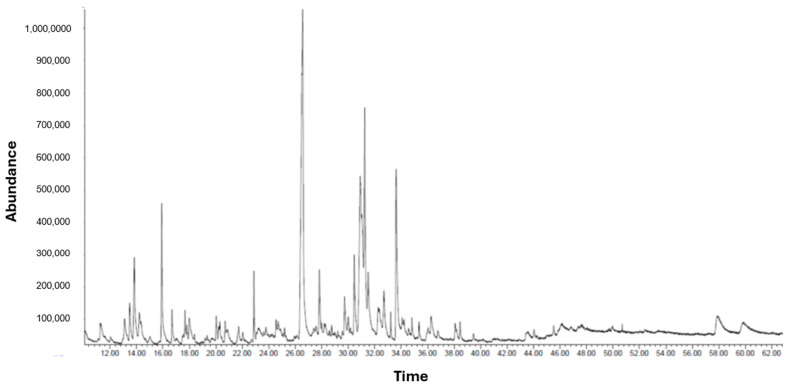
GC–MS chromatogram of the ethanol extract of *Atraphaxis pyrifolia*.

The chromatographic profiles (Figure 5 and Figure 6) support these compositional differences. The chromatogram of *A. virgata* (Figure 5) is characterized by a dominant peak tentatively assigned by library matching to an acetophenone-related compound. This assignment requires further confirmation because the library match probability was below 90%. In contrast, the chromatogram of *A. pyrifolia* (Figure 6) shows multiple peaks of moderate intensity distributed across a wider retention-time range, indicating a more diverse chemical composition.

Overall, the results demonstrate that ultrasound-assisted extraction of CO_2_-extracted biomass yields chemically distinct extracts for each species. The GC–MS profiles indicate clear differences between the two species. *A. virgata* showed a more concentrated chromatographic profile dominated by a major peak tentatively assigned to an acetophenone-related constituent, whereas *A. pyrifolia* showed a broader profile containing putative carbohydrate-derived, phenolic-related, oxygenated, and acetophenone-related constituents. These annotations require confirmation by authentic standards or complementary structural methods before definitive compound-level conclusions can be made. These compositional differences may have important implications for their pharmacological potential and should be further investigated through targeted bioactivity assays and compound isolation. The use of CO_2_-extracted residual biomass is also conceptually important because it demonstrates that the plant meal remaining after lipophilic extraction still contains chemically valuable constituents. This supports a more complete utilization of *Atraphaxis* raw material and is consistent with waste-reducing approaches in plant-based product development [32,33,34,36]. The GC–MS data provide relative peak-area information only and do not represent validated absolute concentrations. Therefore, they are useful for comparative profiling but cannot establish dose–response relationships between specific compounds and the histological effects observed in vivo. Future studies should include validated quantitative analysis of major marker compounds, particularly acetophenone-related constituents, phenolic compounds, and fatty-acid-related metabolites, using authentic reference standards and calibration curves. Because the CO_2_ extract and the hydroethanolic residual-biomass extract were not tested separately in animals, the toxicological findings cannot be attributed to a specific extraction fraction or to individual GC–MS-detected compounds.

### 2.6. LC–MS Analysis of Polyphenolic Compounds in the Ethanol Extract of Atraphaxis virgata

**Figure 7:**
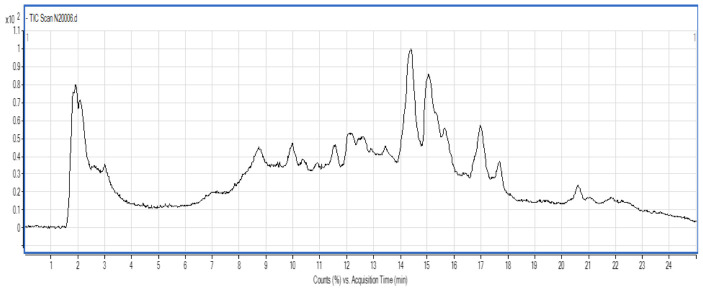
Total ion chromatogram (TIC) of polyphenolic compounds in the *A. virgata* extract obtained by HPLC−MS in negative ionization mode.

Polyphenolic compounds in the ethanol extract obtained from CO_2_-extracted biomass of *A. virgata* were analyzed by high-resolution liquid chromatography coupled with tandem mass spectrometry (LC–MS). Negative ionization mode was used because it is suitable for detecting phenolic acids and flavonoids, which readily form stable deprotonated ions under these conditions. Tentative structural annotation was performed by interpreting MS/MS fragmentation patterns and comparing the results with literature data and spectral databases [14,15,37,38,39]. A total of 19 compounds were tentatively annotated (Table 8) based on accurate mass measurements, retention times, and characteristic fragment ions, in agreement with established LC–MS/MS–QTOF profiling approaches for plant metabolite identification [16,17]. Some of the listed compounds or compound classes have been previously reported in *Atraphaxis* species or related medicinal plants; however, in the present study, they are reported only as tentative annotations because authentic reference standards and NMR confirmation were not used. The tentatively annotated metabolites included phenolic acids, flavonoids, coumarins, and phenylpropanoid derivatives. Representative chromatographic data are presented in Figure 7.

The LC–MS profile of *A. virgata* was dominated by tentatively annotated phenolic acids, flavonoids, coumarins, and phenylpropanoid derivatives. These compound classes are frequently associated in the literature with antioxidant and anti-inflammatory potential [19,20,23,40,41,42]. However, because the present study was based on untargeted profiling rather than compound isolation, validated quantification, or direct bioactivity testing, the biological relevance of these annotations should be interpreted at the compound-class level rather than assigned to individual metabolites.

The total ion chromatogram (TIC) obtained in negative ionization mode (Figure 7) showed a complex multi-component profile of polyphenolic compounds. Multiple elution regions with different peak intensities indicated a wide polarity range, from highly polar phenolic acids to less polar flavonoid aglycones and conjugates. These results support the suitability of LC–MS for comprehensive profiling of plant polyphenols [38,43]. The results are consistent with previous studies on *A. virgata*. For example, Shin et al. [14] reported major polyphenols such as hesperidin, rutin, catechin, gallic acid, epicatechin gallate, kaempferol, and luteolin using HPLC analysis. These compounds belong to the same principal classes identified in the present study, namely phenolic acids and flavonoids, and support the phytochemical richness of this species. These findings indicate that *A. virgata* contains a chemically diverse polyphenolic profile that may support future bioactivity-guided studies. Nevertheless, pharmacological or nutraceutical relevance should be confirmed through targeted biological assays and validated analysis of marker compounds.

Overall, LC–MS analysis indicates that *A. virgata* contains a diverse profile of tentatively annotated polyphenolic compounds, particularly flavonoids and phenolic acids. Because the analysis was qualitative and annotation-based, these data support phytochemical characterization but do not allow direct correlation between individual metabolites and toxicological findings. Future studies should include validated quantitative analysis, compound isolation, and targeted bioactivity testing.

### 2.7. LC–MS-Based Tentative Annotation of Polyphenols in the Ethanolic Extract of Atraphaxis pyrifolia

Mass spectrometric analysis of polyphenolic compounds was performed using a high-resolution quadrupole time-of-flight (QTOF) tandem mass spectrometer coupled to a liquid chromatography system (LC–ESI–QTOF–MS). The analysis was conducted on the ethanolic extract obtained from *A. pyrifolia* residual biomass. Data acquisition was performed in negative ionization mode, which is suitable for phenolic compounds because they readily form deprotonated [M–H]^−^ ions [15,38]. Structural characterization was based on MS/MS fragmentation patterns and comparison with literature data and spectral databases [16,43].

**Figure 8:**
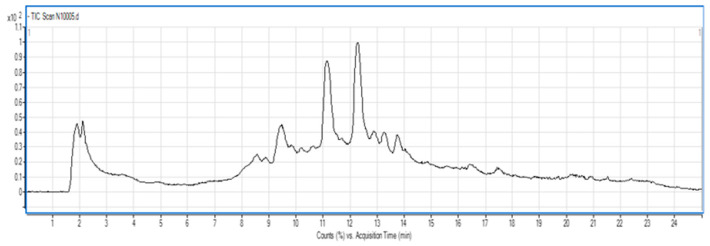
Total ion chromatogram (TIC) of polyphenolic compounds obtained by LC–MS in negative ionization mode for the extract of *A. pyrifolia*.

A range of phenolic compounds was tentatively annotated in the extract, and the results are summarized in Table 9. In total, 17 compounds were tentatively annotated based on accurate mass measurements, characteristic fragment ions, and retention behavior, in agreement with established LC–MS-based metabolite profiling approaches [15,16]. Several annotations are consistent with previous reports on *Atraphaxis* species and related medicinal plants; nevertheless, they remain tentative in this study because reference-standard and NMR confirmation were not performed. The tentatively annotated metabolites included flavonoids (quercetin, luteolin, and kaempferol derivatives), flavan-3-ols (catechin and procyanidins), phenolic acids (gallic acid and dihydrocaffeic acid), coumarins (scopoletin), and lignans. The chromatographic profile of the major polyphenolic constituents in the aerial parts is shown in Figure 8.

The phytochemical profiling results are scientifically relevant because published data on *Atraphaxis* species remain limited. Previous studies have only partially addressed the isolation and structural characterization of compounds from the aerial parts of *A. pyrifolia*. The present work provides a broader evaluation of its chemical profile and offers preliminary evidence for future pharmacological investigation.

Earlier investigations by Abilkassymova et al. [5] using high-resolution ESI–QTOF–MS analysis of *A. pyrifolia* leaves identified several characteristic flavonoids, including 8-acetoxy-3′,4′,5,5′-tetrahydroxy-7-methoxy-3-rhamnopyranosyloxyflavone, pyrifolin, and dehydroxypyrifolin. Related compounds, such as 3,4′,5,7,8-pentahydroxyflavone derivatives, were also reported. These findings are consistent with the present results and indicate both chemical continuity and variability within the genus, supporting phytochemical profiling as a useful tool for chemotaxonomic studies.

The tentative annotation of flavonoids, flavan-3-ols, phenolic acids, coumarins, and lignan-related compounds suggests that the *A. pyrifolia* extract contains compound classes commonly associated with biological activity in the literature [5,20,21,22,23,40,41,42]. To avoid overinterpretation, these findings are discussed as evidence of phytochemical richness rather than proof of specific pharmacological effects. Confirmation using authentic standards, compound isolation, validated quantification, and targeted bioactivity testing is required before biological effects can be assigned to individual metabolites.

The chromatographic profile obtained for the polyphenolic fraction in negative ionization mode (Figure 8) was characterized by numerous well-resolved peaks, indicating a complex multi-component extract composition. Multiple elution regions with different peak intensities reflected a broad polarity range, from highly polar phenolic acids to less polar flavonoid aglycones and conjugates. These results confirm the effectiveness of the chromatographic separation and the suitability of LC–MS for comprehensive plant polyphenol profiling [38,43].

Overall, LC–MS profiling revealed a chemically diverse polyphenolic profile in *A. pyrifolia* and confirmed the suitability of the applied method for comparative phytochemical characterization. However, the analysis remains qualitative and annotation-based. Because absolute concentrations of key marker compounds were not determined, the phytochemical data cannot be directly correlated with the dose-dependent histological changes observed in the acute toxicity experiment.

### 2.8. Acute Toxicity Assessment of Hydroethanolic Extracts of Atraphaxis pyrifolia and Atraphaxis virgata

A key criterion for obtaining high-quality plant-derived products is the use of standardized raw materials together with non-clinical safety evaluation of phytosubstances [44,45]. Non-clinical evaluation of herbal medicinal products includes assessment of acute, subacute, and chronic toxicity. For topical dosage forms, local irritant and allergenic effects should also be evaluated, together with pharmacological activity.

The study was conducted at the Scientific Center for Anti-Infective Drugs, Laboratory of Pharmacology and Toxicology, Almaty, Kazakhstan.

The aim was to evaluate acute toxicity and determine the median lethal dose (LD_50_) of *A. virgata* and *A. pyrifolia* extracts after oral administration in mice. Acute toxicity was assessed using the LD_50_ value, defined as the dose causing death in 50% of experimental animals, and was used to classify the toxicity of the tested substances.

The toxicity class of the tested extracts was determined in accordance with the Order of the Ministry of Health of the Republic of Kazakhstan dated 4 February 2021 (No. KR DSM-15) [44] and modified Organisation for Economic Co-operation and Development (OECD) principles for acute oral toxicity [45]. Under this classification approach, substances are categorized according to oral LD_50_ values. Because no mortality was observed after oral administration of the extracts at doses up to 2000 mg/kg, the LD_50_ was considered greater than 2000 mg/kg. This result supports a low acute oral toxicity classification under the tested single-dose conditions. Nevertheless, interpretation was not based solely on mortality; histopathological findings were also considered to evaluate possible tissue-level effects at higher doses.

The acute toxicity of the hydroethanolic extracts of *A. virgata* and *A. pyrifolia* was evaluated in outbred white mice of both sexes, weighing 22–27 g. Animals were divided into four groups (n = 5 per group), including a control group. The extracts were administered orally at 300, 500, 1000, and 2000 mg/kg under fasting conditions.

#### 2.8.1. Observation of Experimental Animals

Animal observations after single oral administration of *A. virgata* and *A. pyrifolia* extract solutions at different dose levels are presented in Table 10.

No mortality or treatment-related adverse clinical signs were observed in mice after single oral administration of hydroethanolic extracts of *A. virgata* and *A. pyrifolia* at doses of 300, 500, 1000, and 2000 mg/kg during the 14-day observation period. All animals remained in normal physiological condition, and no signs of acute intoxication, behavioral abnormality, or visible changes in appearance were recorded. Under OECD Test Guideline 423, absence of mortality at the 2000 mg/kg limit dose indicates that the oral LD_50_ exceeds 2000 mg/kg. Although this finding supports low acute toxicity, histological data revealed mild dose-dependent tissue alterations at high exposure levels [45]. These findings are consistent with recent toxicity studies of other polyphenol-rich or hydroethanolic plant extracts. For example, a polyphenolic extract of Flourensia cernua tested under OECD 423 caused no mortality at 300 or 2000 mg/kg, although transient toxicity signs were noted shortly after dosing [46]. A phenolic-rich extract evaluated in Plants also caused no mortality after oral administration at 2000 mg/kg during short- and longer-term observation periods [47]. Similarly, a 2025 study of *Piper marginatum* hydroethanolic extract conducted according to OECD 423 reported no mortality or significant behavioral changes in mice [48]. Together, these studies support the view that phenolic- and flavonoid-rich plant extracts often show a favorable acute oral safety profile, although individual extracts may differ depending on composition and concentration [46,47,48].

The low acute toxicity observed for *A. virgata* and *A. pyrifolia* can be discussed in relation to the phytochemical profiles described in Section 2.5, Section 2.6 and Section 2.7. However, because the chemical analysis was not absolute or validated quantitatively, direct correlations between individual constituents and toxicological outcomes cannot be established. GC-MS and LC-MS analyses showed that both extracts contained abundant polyphenolic constituents, including flavonoids, phenolic acids, coumarin derivatives, and related phenylpropanoids. In *A. virgata*, the detected metabolites included quercetin derivatives, luteolin 6-C-glucoside, apigenin, nepetin, caffeoylquinic acid derivatives, and scopoletin-type compounds. In *A. pyrifolia* catechin, procyanidin, luteolin, quercetin and kaempferol glycosides, gallic acid, dihydrocaffeic acid, and related phenolic constituents were detected. These compound classes are widely associated with antioxidant, anti-inflammatory, antimicrobial, and cytoprotective activities and are generally considered to have relatively low intrinsic acute toxicity at nutraceutical or phytotherapeutic exposure levels [5,49].

At the same time, these results should be interpreted cautiously. Absence of mortality and overt clinical signs after a single oral dose does not exclude subacute, subchronic, organ-specific, reproductive or genotoxic effects. OECD 423 is intended primarily for hazard identification under acute exposure conditions rather than full toxicological characterization [45]. Nevertheless, the combination of a polyphenol-rich composition and an LD_50_ above 2000 mg/kg provides an encouraging initial safety signal and supports further pharmacological and nutraceutical investigation of *Atraphaxis* extracts [5,45,49].

#### 2.8.2. Body Weight Monitoring and Organ Weight Analysis

Body weight changes in mice after single oral administration of *A. virgata* and *A. pyrifolia* aqueous extracts are presented in Table 11. Across all experimental groups (300–2000 mg/kg), body weight increased gradually during the 14-day observation period. No clear dose-related decrease in body weight was observed.

The positive weight gain indicates normal physiological development and no overt systemic toxicity. Body weight is a sensitive indicator of general health status in toxicological studies; therefore, the absence of weight loss or growth suppression supports the low acute toxicity profile of the tested extracts.

These findings are consistent with previous acute toxicity studies reporting no significant body weight changes after administration of plant extracts at comparable dose ranges [45,46,47,48,50]. Absolute organ weights measured at the end of the experimental period are presented in Table 12. Organ weight values remained within a comparable range across treated groups and showed no clear dose-related pattern.

The absence of clear dose-related changes in organ weights suggests that the extracts did not cause marked organ-level toxicity under the conditions of this acute study. The liver and kidneys, key organs involved in xenobiotic metabolism and excretion, remained within comparable physiological ranges. These observations are consistent with previous acute toxicity studies of plant-derived extracts, in which no significant changes in organ weights or clear signs of hepatotoxicity or nephrotoxicity were observed [51,52].

The absence of clear adverse effects on body weight and organ weight may be considered together with the phytochemical composition described in Section 2.5, Section 2.6 and Section 2.7. However, because marker compounds were not quantified using validated methods, these findings should be interpreted as supportive observations rather than evidence of a direct chemical–toxicological relationship. The identified compound classes, including flavonoids, phenolic acids, and coumarins, may contribute to the overall biological profile of the extracts. However, the present study does not allow the contribution of individual compounds to physiological stability or tissue-level effects to be determined.

Overall, these results indicate no clear adverse effect on body weight or organ weight after single-dose oral administration of *A. virgata* and *A. pyrifolia* extracts at doses up to 2000 mg/kg. However, because histological alterations were observed at higher doses, these findings should be interpreted as supportive evidence of low acute oral toxicity rather than proof of complete organ safety or suitability for prolonged pharmacological use.

#### 2.8.3. Macroscopic Examination of Organs

Macroscopic examination after single oral administration of *Atraphaxis virgata* and *Atraphaxis pyrifolia* aqueous extracts at doses of 300, 500, 1000, and 2000 mg/kg revealed no major pathological alterations in internal organs. Across all groups, the general anatomical integrity of the examined organs was preserved. At higher doses (1000 and 2000 mg/kg), slight thyroid enlargement was observed. This change was not accompanied by visible structural abnormalities, discoloration, or systemic clinical signs, suggesting that it may represent a non-adverse adaptive or physiological response rather than overt toxicity. However, histopathological evaluation would be required to exclude microscopic alterations.

In all treated groups, the axillary and inguinal lymph nodes and prostate gland remained unchanged. The thoracic cavity contained no fluid accumulation. Mild congestion was observed in certain lung lobes; however, light, slightly foamy fluid in the bronchi and the ability of lung tissue to float in liquid indicated preserved aeration and normal respiratory structure. The heart showed normal morphology, with clearly visible coronary vessels, no thickening of the left ventricular wall, and intact cardiac valves. The thymus and thyroid glands were located in their typical anatomical positions, with only mild thyroid enlargement. The diaphragm was intact. The liver had a characteristic dark cherry coloration without evidence of hemorrhage or necrosis, and the kidneys maintained a normal light-brown appearance. The spleen retained its typical folded structure, and the stomach contained normal contents without visible lesions. Reproductive organs and the oral cavity showed no visible abnormalities.

Overall, the absence of major macroscopic pathological changes suggests that the tested extracts did not cause visible organ damage after acute oral administration. However, this finding should be interpreted together with the histopathological results, which revealed mild to moderate tissue-level alterations at higher doses. These observations are consistent with the body weight and organ weight data (Section 2.8.1 and Section 2.8.2) and further support a low acute toxicity profile under the conditions of this single-dose study.

The observed acute safety profile may be related to the phytochemical composition characterized in Section 2.5, Section 2.6 and Section 2.7, where the extracts were shown to contain diverse polyphenolic constituents, including flavonoids, phenolic acids, and coumarins. These classes of secondary metabolites are widely associated with low acute toxicity and cytoprotective properties, which may contribute to the maintenance of organ integrity under experimental conditions. The present findings are consistent with previous toxicological studies of plant-derived extracts that reported no significant macroscopic alterations in internal organs after acute exposure at comparable doses [51,52]. Macroscopic examination is a standard component of acute toxicity assessment and provides preliminary evidence of organ integrity, although it should be complemented by histopathological analysis for a more complete evaluation [45].

#### 2.8.4. Histological Evaluation

Histological analysis was performed according to SOP-PHT-020 and was initiated after macroscopic changes were observed in internal organs. Microscopic examination of tissue samples from animals treated with *A. virgata* and *A. pyrifolia* aqueous extracts at doses of 1000 and 2000 mg/kg revealed consistent dose-related morphological alterations in several organs. Representative micrographs are provided in the Supplementary Materials (Figures S1 and S2).

Microscopic observations (Figures S1 and S2) showed organ-specific changes. In *A. virgata*-treated animals (Figure S1), the spleen showed diffuse edema of the red pulp, accumulation of siderophages and early fibrotic changes. Renal sections showed narrowing of proximal and distal tubules and mild glomerular alterations, including mesangial hypercellularity. Cardiac tissue showed dystrophic changes in cardiomyocytes, whereas liver sections showed dilation of Disse spaces and activation of stellate cells.

In *A. pyrifolia*-treated animals (Figure S2), similar but slightly more pronounced alterations were observed. These included epithelial changes in renal tubules, focal lymphocytic infiltration in the pancreas, and erythrophagocytosis in the spleen. Liver sections also showed hepatocyte necrobiosis and micro- to macrovesicular degeneration. Despite these findings, the overall tissue architecture remained largely preserved.

The histopathological changes were generally mild to moderate and did not indicate extensive or irreversible tissue damage. In the liver, slight hepatocellular alterations, including mild cellular swelling and limited structural disorganization, were observed without extensive necrosis or inflammatory infiltration. Renal tissue showed minor tubular epithelial changes, while glomerular structures were largely preserved. Mild vascular congestion and limited interstitial changes were observed in the lungs, consistent with the macroscopic findings. Other organs retained normal histoarchitecture.

From a toxicological perspective, these findings suggest low acute toxicity because no mortality was observed at doses up to 2000 mg/kg (LD_50_ > 2000 mg/kg). However, histopathological evaluation revealed mild to moderate dose-dependent alterations in liver and kidney tissues at higher doses. These results are consistent with previous studies on plant-derived extracts, where mild histological changes at high doses occurred without significant functional impairment [51,52]. The observed effects may reflect metabolic adaptation in detoxification organs such as the liver and kidneys. The findings can also be discussed in relation to the phytochemical composition described in Section 2.5, Section 2.6 and Section 2.7, but only at the whole-extract and compound-class levels. Because absolute concentrations of major constituents were not determined, the histological changes cannot be attributed to specific flavonoids, phenolic acids, acetophenone-related compounds, or other individual metabolites. The dose-dependent tissue alterations are therefore interpreted as responses to high-dose exposure to the whole hydroethanolic residual-biomass extract rather than as toxicity of a specific compound or extraction fraction. These morphological changes were dose-dependent but were not associated with lethality, indicating subclinical effects rather than acute systemic toxicity. Importantly, they also show that high-dose exposure was not biologically neutral. Because the liver and kidneys are major organs involved in xenobiotic metabolism and elimination, the observed alterations may represent adaptive or stress-related responses to high-dose extract exposure. These findings support a cautious interpretation of safety and highlight the need for repeated-dose toxicity studies with biochemical, hematological, and histopathological endpoints.

Overall, histopathological evaluation suggests that lower doses did not produce major tissue disruption under the acute study conditions, whereas higher doses induced mild to moderate organ-level changes. Therefore, the results support a low acute toxicity classification but do not establish complete toxicological safety. This finding is important because it shows that acute safety assessment should not rely only on mortality or LD_50_ values. The combination of survival data and histological evaluation provides a more cautious and biologically informative safety profile, indicating that further subacute and chronic toxicity studies are required before practical pharmaceutical or nutraceutical applications are considered. Because the acute toxicity experiment included five mice per group, the quantitative body weight and organ weight data should be interpreted as descriptive supportive evidence. Future studies with larger sample sizes and longer subacute or chronic exposure periods are required to confirm the safety profile with stronger statistical power. A direct mechanistic linkage between extraction selectivity and toxicological outcome will require separate toxicological evaluation of the CO_2_ extract, the hydroethanolic residual-biomass extract, and purified or standardized marker fractions. Such a design would help determine whether tissue-level effects are mainly associated with lipophilic CO_2_-soluble compounds, polar polyphenolic constituents, or combined matrix effects of the whole extract.

Further studies are required to define the toxicological safety margin of these extracts. In particular, 28-day subacute and 90-day subchronic repeated-dose toxicity studies are needed to establish NOAEL values and to determine whether the observed liver and kidney alterations are reversible, adaptive, or adverse. Future investigations should also include targeted hepatotoxicity and nephrotoxicity biomarkers, such as serum ALT, AST, ALP, bilirubin, urea, creatinine, electrolyte balance, urinalysis, oxidative stress markers, and extended histopathological scoring. Basic genotoxicity screening would also be relevant if pharmaceutical or nutraceutical development is considered.

## 3. Materials and Methods

### 3.1. Plant Material

The aerial parts of *A. pyrifolia* were collected from the slopes of the Kokpek Ridge, Almaty region, Kazakhstan (43°26′54″ N, 78°40′30″ E), while *A. virgata* was collected from the Aksai Gorge in the Northern Tien Shan Mountains (Zailiyskiy Alatau, Kazakhstan), Almaty region (43°07′20.5″ N, 76°47′54.59″ E). Sampling was performed in June 2022 during the flowering stage.

The plant materials were taxonomically identified by a qualified botanist. Voucher specimens were deposited at the Herbarium of Farabi University, Almaty, Kazakhstan under voucher numbers AV2022-07 and AP2022-07.

The collected plant materials were air-dried under ambient conditions at room temperature (25 ± 5 °C) and relative humidity of 60 ± 5% in a shaded, well-ventilated area for 21 days using drying frame. During drying, leaves, flowers, and stems were turned every two days to ensure uniform moisture removal. The dried materials were examined and cleaned to remove foreign impurities, including soil particles, dust, and insects.

All experimental procedures were carried out at the Department of Chemical Technology of Organic Substances, Natural Compounds and Polymers, Faculty of Chemistry and Chemical Technologies, Farabi University.

### 3.2. Anatomical and Morphological Study of Leaves and Stems of Atraphaxis virgata and Atraphaxis pyrifolia

Anatomical analyses were performed using both fixed and freshly collected plant material following established protocols [53,54,55] and recent methodological approaches [56,57]. Transverse sections of stems and leaves were prepared manually and using a freezing microtome (TOS-2, Moscow, Russia).

Air-dried plant material was rehydrated in a mixture of 70% ethanol, glycerol, and distilled water (1:1:1, Straus–Fleming solution). Intact fully developed leaves from the middle portion of the shoots were selected for analysis. Epidermal preparations and transverse sections were examined. Temporary slides, including epidermal peels, squash preparations, and transverse sections, were prepared according to standard methods. Samples were cleared with glycerol. Epidermal preparations were obtained by boiling leaves in 10% potassium hydroxide (KOH) solution.

Morphological characterization was conducted using standard methods and focused on the structural features of stems, leaves, and sepals [58,59]. Microscopic observations were performed using a light microscope (MC 300, Micros Produktions- und HandelsgmbH, Vienna, Austria) at magnifications of ×70 and ×100. Morphometric measurements were made using an ocular micrometer (MOV-1-15, LOMO, Saint Petersburg, Russia; objective ×9, eyepiece ×10.7). Microphotographs were captured using a CAM V400/1.3M digital camera (jProbe Co., Ltd., Yokohama, Japan). All analyses were conducted at the Laboratory of Plant Anatomy and Morphology, Farabi University, Almaty, Kazakhstan. During stem characterization, features visible in transverse sections were examined in detail. At low magnification (×10), the primary cortex, vascular cylinder (stele), overall outline, cell morphology, tissue structure, and distribution of vascular elements were described.

Leaf sections were prepared from the central part of the lamina. Plants at the generative stage were selected, with emphasis on middle-aged generative individuals representing the main raw material source for the studied species. For biometric analysis, five representative anatomical sections or microscopic fields were measured for each organ and species. Samples were selected from intact, fully developed, and morphologically normal leaves and stems at comparable developmental stages. Therefore, the biometric data in Table 1, Table 2, Table 3 and Table 4 should be interpreted as descriptive anatomical measurements of representative samples rather than population-level estimates.

Specimens were prepared and described according to standard methods in plant anatomy [60]. External and internal features were characterized and performed in accordance with the requirements of the State Pharmacopoeias [53,61,62,63].

### 3.3. Subcritical Carbon Dioxide Extraction

Extraction was carried out under subcritical conditions using UUPE-5L laboratory carbon dioxide flow extraction unit (Russia) at ZHANAFARM LLP (Manufacturer of Medicinal Products, Almaty, Kazakhstan). Liquid carbon dioxide (CO_2_) was used as the extractant in accordance with GOST 8050-85 [31].

The following plant materials were extracted: *A. virgata* aerial parts, *A. pyrifolia* aerial parts, and *A. pyrifolia* stems. Before extraction, the plant material was milled using a KDU-2 grinder (LLC “AgroPostavka”, Nizhny Novgorod, Russia) to a particle size of 3–5 mm to increase the specific surface area and improve extraction efficiency.

The extraction parameters and yields of carbon dioxide extracts from *A. virgata* and *A. pyrifolia* are presented in Table 13.

### 3.4. Ultrasound-Assisted Extraction of Plant Meal (A. virgata and A. pyrifolia)

Air-dried and milled plant meal, representing the solid residue after CO_2_ extraction was extracted with 70% (*v*/*v*) ethanol for 120 min at 35–40 °C using a solid-to-solvent ratio of 1:8 (*w*/*v*). The amounts of plant meal were 660 g for *A. virgata* and 1380 g for *A. pyrifolia*. Extraction was carried out in an ultrasonic bath (Sapphire, Moscow, Russia) operating at 35 kHz.

The extracts were filtered and concentrated to dryness under reduced pressure using a rotary evaporator (Rotavapor^®^ R-300, Büchi Labortechnik AG, Flawil, Switzerland). The yield of extractive substances was determined gravimetrically.

### 3.5. GC–MS Analysis of the Component Composition of Ethanol Extracts of A. virgata and A. pyrifolia

The chemical composition of ethanol extracts obtained from the aerial parts of *A. virgata* and *A. pyrifolia* was analyzed by gas chromatography–mass spectrometry (GC–MS) using an Agilent 7890B gas chromatograph coupled to a 5977A mass selective detector (Agilent Technologies, Santa Clara, CA, USA). Before analysis, 0.5 g of each extract was dissolved in 2 mL of hexane, and an aliquot was injected into the GC system.

The injection volume was 0.4 μL, and the injector temperature was 240 °C. The injector was operated in splitless mode. Chromatographic separation was performed on a DB-17 ms capillary column (30 m × 0.25 mm i.d., 0.5 μm film thickness; Agilent Technologies, Santa Clara, CA, USA) using helium as the carrier gas at a constant flow rate of 1 mL/min.

The oven temperature was programmed from 40 °C to 280 °C at 5 °C/min and then held at 280 °C for 15 min. Total analysis time was 63 min. Mass spectrometric detection was performed in electron ionization (EI) mode at 70 eV, with SCAN acquisition over an *m*/*z* range of 34–800. The ion source and quadrupole temperatures were 230 °C to 150 °C, respectively.

System control, data acquisition, and processing were performed using Agilent MSD ChemStation software (version E.01.00; Agilent Technologies, Santa Clara, CA, USA). Data analysis included determination of retention times and peak areas and interpretation of mass spectra obtained from the mass selective detector. Compound identification was performed by comparison with the Wiley 7th edition and NIST 02 mass spectral libraries. Authentic reference standards were not used for GC–MS confirmation. Therefore, GC–MS results are reported as tentative library-based annotations rather than confirmed compound identifications. Library match probabilities below 90% were considered low-confidence annotations and were interpreted cautiously, preferably at the putative compound-class level. Relative abundance values are reported as relative peak areas and do not represent absolute concentrations.

### 3.6. LC–MS/MS Analysis of Polyphenols in Ethanol Extracts of Plant Meal from A. virgata and A. pyrifolia

Polyphenolic compounds were analyzed by high-resolution tandem mass spectrometry using an Agilent 6520B quadrupole time-of-flight (QTOF) LC–MS system (Agilent Technologies, Santa Clara, CA, USA). Analyses were performed using an electrospray ionization (ESI) source in negative ion mode. The drying gas flow rate was 5 L/min, drying gas temperature was 300 °C, skimmer voltage was 20 V, and fragmentor voltage was 125 V. Mass spectra were acquired over *m*/*z* 100–2000 in MS mode and *m*/*z* 50–2000 in targeted MS/MS mode. Collision energies were set at 35 and 50 eV.

Samples were introduced using an Agilent 1200 Series HPLC system (Agilent Technologies, Santa Clara, CA, USA) equipped with a Zorbax SB-C18 column (3 μm, 0.5 × 150 mm; Agilent Technologies). The mobile phase consisted of 0.1% formic acid in water (A) and acetonitrile containing 0.1% formic acid (B). Chromatographic separation was performed using an Agilent 1260 Cap Pump at a flow rate of 15 μL/min under the following gradient: 0–5 min, 20%; 20 min, 25%; 25 min, 30%; 25.1–30 min, 60%; and 35 min, 20%. Solvents were degassed using a 1260 µ-degasser. A 1 μL sample injection was performed using an Agilent Micro WPS autosampler.

Compound characterization was performed by interpreting MS/MS spectra and comparing them with literature data. Identification was supported by public databases, including ChemSpider (http://www.chemspider.com, accessed on 15 February 2026), SciFinder Scholar (https://scifinder.cas.org, accessed on 17 February 2026), KEGG Ligand (http://www.genome.jp/kegg/ accessed on 17 February 2026), and Phenol-Explorer (http://www.phenol-explorer.eu accessed on 20 February 2026). Authentic reference standards were not analyzed, and NMR confirmation was not performed for LC–MS assignments. Therefore, LC–MS results are reported as tentative structural annotations rather than confirmed compound identifications. Biological interpretation was made primarily at the compound-class level unless the annotation was strongly supported by accurate mass, diagnostic MS/MS fragments, and consistency with literature data.

### 3.7. Acute Toxicity Assessment of Aqueous–Ethanolic Extracts of A. pyrifolia and A. virgata

The study was conducted at the Scientific Center for Anti-Infective Drugs, Laboratory of Pharmacology and Toxicology, Almaty, Kazakhstan. Before experimentation, animals underwent a two-week acclimatization period and were maintained on a standard laboratory diet under vivarium conditions.

Acute toxicity was evaluated using outbred white mice of both sexes with body weights of 22 g ± 10%. Animals were randomly divided into four groups (n = 5 per group), including a control group. Extracts were administered orally as a single dose at 300, 500, 1000, or 2000 mg/kg body weight under fasting conditions. The observation period lasted 14 days.

The experimental protocol included daily monitoring of general condition, body weight measurements at three time points, and necropsy of all surviving animals at the end of the experiment. Macroscopic examination was performed, followed by determination of relative organ weights and histological analysis of organs with visible pathological changes.

#### 3.7.1. Preparation and Administration of Test Solutions

The test substances were administered as a single oral dose, corresponding to the intended clinical route of administration and complying with the regulations of the Acting Minister of Health of the Republic of Kazakhstan (Order No. RK DCM-15, dated 4 February 2021; registered with the Ministry of Justice of the Republic of Kazakhstan on 9 February 2021, No. 22167).

Working solutions of *A. pyrifolia* and *A. virgata* extracts were prepared by dissolving accurately weighed extract portions in distilled water to obtain target doses of 300, 500, 1000, and 2000 mg/kg body weight. The extract amount was adjusted according to animal body weight in each experimental group. Solutions were prepared immediately before administration and were not stored.

The dose levels, group allocation, and number of animals are presented in Table 14.

After 14 days of observation (day 15), the animals were euthanized and subjected to autopsy and macroscopic examination. Internal organs, including the liver, lungs, spleen, kidneys, and heart, were excised and weighed.

#### 3.7.2. Analysis of Somatic Parameters

Changes in external somatic characteristics, including fur condition, coloration of the oral mucosa, and other clinical signs, were recorded during daily observations throughout the experimental period. Observation parameters, toxic effects detected during the acute toxicity study, and criteria for their assessment were documented according to SOP-PHT-021.

#### 3.7.3. Body Weight Analysis

Body weight was measured weekly: before the start of the experiment, during the study, and at termination. All measurements were recorded in grams.

#### 3.7.4. Euthanasia of Animals

On the final day of the experiment (day 15), animals were euthanized under isoflurane anesthesia according to SOP-PHT-016.

#### 3.7.5. Macroscopic Analysis

Macroscopic examination was performed according to SOP-PHT-019 “Macroscopic Examination of Laboratory Animal Internal Organs”. The assessment included evaluation of the external body surface, natural orifices, regional lymph nodes, internal organs, and tissues.

#### 3.7.6. Organ Weight Analysis

After autopsy, internal organs (liver, lungs, spleen, kidneys, and heart) were excised and weighed. Organ weights were recorded for animals from all experimental groups during scheduled necropsy.

#### 3.7.7. Histological Analysis of Organs

When visible pathological changes were observed in internal organs, histological examinations were performed according to SOP-PHT-020.

## 4. Conclusions

The main novelty of this work is the direct comparison of both species using the same extraction protocol, analytical methods, and toxicity assessment design, with special emphasis on the chemical value of CO_2_-extracted residual biomass. This study establishes an integrated framework for the pharmacognostic, phytochemical, and preliminary toxicological evaluation of *A. virgata* and *A. pyrifolia*. The combined anatomical and chemical data support differentiation of these two under-investigated xerophytic species and provide baseline information for botanical authentication and future standardization of raw plant material.

Sequential processing of CO_2_-extracted residual biomass showed that the remaining plant meal retains chemically valuable constituents, supporting more complete utilization of *Atraphaxis* biomass. GC–MS and LC–MS profiling revealed species-specific metabolite patterns; however, because authentic standards, NMR confirmation, and validated absolute quantification were not used, the reported compounds should be interpreted as tentative metabolite annotations.

The acute toxicity findings support a low acute oral toxicity classification under the tested single-dose conditions. However, dose-related histological alterations indicate that high-dose exposure is not biologically neutral. Therefore, these data should be regarded as preliminary acute toxicity evidence rather than proof of comprehensive safety. Future studies should include validated marker-compound quantification, reference-standard confirmation, fraction-specific bioactivity testing, and 28-day and 90-day repeated-dose toxicity studies to establish NOAEL values and clarify the toxicological relevance of the observed hepatic and renal changes.

## Figures and Tables

**Table 1:** Biometric characteristics of representative leaf samples of *Atraphaxis pyrifolia* Bunge (n = 5).

Sample	Lower Epidermis (µm)	Upper Epidermis (µm)	Leaf Thickness (µm)	Mesophyll Thickness (µm)	Palisade Mesophyll (µm)	Spongy Mesophyll (µm)	Vascular Bundle Diameter (µm)	Druse Diameter (µm)
1	0.360	0.540	22.540	20.671	8.462	9.540	245.06	8.245
2	0.560	0.396	23.160	15.236	9.003	8.280	245.06	4.891
3	0.653	0.469	19.427	16.102	7.900	7.120	245.06	6.738
4	0.436	0.512	20.796	13.020	5.386	6.890	245.06	5.389
5	0.558	0.495	21.021	14.126	6.631	5.920	245.06	3.637
Mean	0.513	0.482	21.389	15.831	7.476	7.550	245.06	5.780

**Table 2:** Biometric characteristics of representative stem samples of *Atraphaxis pyrifolia* Bunge (n = 5).

Sample	Periderm Thickness (µm)	Collenchyma Thickness (µm)	Cambial Zone Thickness (µm)	Primary Cortex Parenchyma (µm)	Central Cylinder Diameter (µm)	Xylem Vessel Diameter (µm)
1	0.695	0.502	1.695	15.535	340.39	0.407
2	1.102	0.728	1.891	16.120	340.39	0.216
3	1.086	0.720	1.738	12.601	340.39	0.474
4	1.389	0.389	1.389	14.468	340.39	0.355
5	1.180	0.428	1.637	13.017	340.39	0.238
Mean	1.090	0.553	1.670	14.348	340.39	0.338

**Table 3:** Biometric characteristics of representative leaf samples of *Atraphaxis virgata* (Regel) Krasn. (n = 5).

Sample	Lower Epidermis (µm)	Upper Epidermis (µm)	Leaf Thickness (µm)	Mesophyll Thickness (µm)	Palisade Mesophyll (µm)	Spongy Mesophyll (µm)	Vascular Bundle Diameter (µm)	Druse Diameter (µm)
1	0.542	0.320	11.162	8.658	5.401	3.060	71.915	0.926
2	0.425	0.285	10.085	8.472	4.861	4.280	71.915	0.867
3	0.320	0.470	11.420	9.721	5.040	3.120	71.915	0.438
4	0.520	0.501	12.503	7.281	6.120	5.002	71.915	0.549
5	0.290	0.352	13.620	6.889	3.590	3.200	71.915	0.620
Mean	0.419	0.386	11.758	8.204	5.002	3.732	71.915	0.680

**Table 4:** Biometric characteristics of representative stem samples of *Atraphaxis virgata* (Regel) Krasn. (n = 5).

Sample	Periderm Thickness (µm)	Collenchyma Thickness (µm)	Cambial Zone Thickness (µm)	Primary Cortex Parenchyma (µm)	Central Cylinder Diameter (µm)	Xylem Vessel Diameter (µm)
1	0.510	0.401	1.720	14.120	325.250	0.501
2	1.202	0.610	1.685	13.210	325.250	0.320
3	1.089	0.528	1.598	12.702	325.250	0.298
4	1.471	0.345	1.445	15.320	325.250	0.449
5	1.150	0.496	1.320	11.200	325.250	0.297
Mean	1.084	0.476	1.554	13.310	325.250	0.373

**Table 5:** Parameters of subcritical CO_2_ extraction of *Atraphaxis* species.

Plant Material (Aerial Parts)	Extraction Mass (g)	Pressure (kgf/cm^2^)	Temperature (°C)	Extraction Time (h)	Extract Yield (g)	Yield (%)
*Atraphaxis virgata*	670	57–65	18–23	8	5	7.46
*Atraphaxis pyrifolia*	1400	57–65	18–23	8	13	9.30

**Table 6:** GC–MS library-based tentative annotations of the ethanol extract obtained by ultrasound-assisted extraction from Atraphaxis virgata CO_2_-extracted meal.

No.	Retention Time (min)	Tentative Annotation/Chemical Class	Library Match Probability (%)	Relative Peak Area (%)
1	12.122	Glycerin	92.2	10.34
2	13.488	1,3-Propanediol	80.3	0.45
3	13.832	Maltol	73.0	0.70
4	14.192	2-Methoxyphenol	94.7	5.31
5	15.904	4H-Pyran-4-one, 2,3-dihydro-3,5-dihydroxy-6-methyl-	92.6	1.04
6	16.681	Cyclopropyl carbinol	78.6	0.27
7	17.536	(E)-2-Octen-1-ol	65.3	0.09
9	17.952	Coumaran	87.5	17.58
10	20.688	2-Methoxy-4-vinylphenol	90.8	1.14
11	23.070	Oxiraneethanol derivative (acetate)	71.6	0.48
12	23.286	Desulphosinigrin	60.0	0.29
13	23.975	Trimethylcyclohexadienyl ketone derivative	78.5	0.10
14	24.336	Ethyl α-D-glucopyranoside	79.0	0.22
15	24.528	Vanillin	87.7	0.74
16	24.920	2-Methyl-2-pentenal	81.3	0.64
17	25.639	Dimethyl phthalate	92.8	0.18
18	26.569	Sucrose	80.0	2.79
19	26.884	Methyl vanillate	78.8	0.12
20	27.390	Benzofuranone derivative	90.9	0.22
21	27.860	D-Allose	86.0	0.30
22	28.986	Allylbenzodioxol derivative	70.1	0.53
23	29.816	Ethyl α-D-glucopyranoside	74.5	0.50
24	30.555	Fructose	71.1	1.79
25	31.323	α-Amino-3′-hydroxy-4′-methoxyacetophenone	81.0	45.26
26	32.256	n-Hexadecanoic acid	80.6	1.82
27	32.669	Methyl homovanillate	86.3	1.34
28	35.972	Propanamide derivative	65.9	1.53
29	49.055	10-Nonadecanol	90.2	4.02

Note: GC–MS assignments were based on comparison with Wiley 7th Edition and NIST’02 mass spectral libraries. Authentic reference standards were not used; therefore, the compounds are reported as tentative library-based annotations. Entries with library match probabilities below 90% should be interpreted as putative annotations or chemical-class assignments rather than confirmed compounds.

**Table 7:** GC–MS library-based tentative annotations of the ethanol extract obtained by ultrasound-assisted extraction from *Atraphaxis pyrifolia* CO_2_-extracted meal.

No.	Retention Time (min)	Tentative Annotation/Chemical Class	Library Match Probability (%)	Relative Peak Area (%)
1	11.246	4-Hydroxy-2-cyclohexen-1-one	73.9	1.17
2	13.109	1,4-Dimethylpiperazine	71.3	1.31
3	13.480	1,3-Propanediol	80.5	1.76
4	13.830	Maltol	74.4	3.93
5	14.204	2-Methoxyphenol	80.3	1.17
6	15.899	4H-Pyran-4-one, 2,3-dihydro-3,5-dihydroxy-6-methyl-	92.9	4.35
7	16.677	Cyclopropyl carbinol	79.0	1.14
8	17.803	1,3-Dioxane-4-methanol derivative	68.1	0.31
9	17.978	2,3-Dihydrobenzofuran	81.5	1.36
10	20.021	Tropine	66.3	0.85
11	20.218	5-Hydroxymethylfurfural	87.3	0.37
12	20.298	Dodecadienone derivative	71.0	0.49
13	20.693	4-Hydroxy-2-methylacetophenone	78.9	0.61
14	21.651	Isosorbide	82.3	0.20
15	21.715	D-Galactose oxime	63.5	0.46
16	22.871	2,6-Dimethoxyphenol	72.9	1.90
17	26.548	Sucrose	79.0	25.39
18	27.815	Levoglucosan	90.7	2.59
19	29.723	Ethyl α-D-glucopyranoside	86.4	1.62
20	30.457	Fructose	74.2	3.37
21	30.896	3-Deoxy-D-mannoic lactone	74.4	16.11
22	31.242	Cyclohexanetetrol derivative	77.3	9.73
23	31.498	Guanosine	69.3	3.25
24	32.258	n-Hexadecanoic acid	73.1	2.26
25	32.695	4-(3-Hydroxy-1-propenyl)-2-methoxyphenol	81.7	1.72
26	33.206	Diisobutyl phthalate	89.2	0.70
27	33.614	2′,6′-Dihydroxy-4′-methoxyacetophenone	95.9	8.28
28	34.807	Pyrrolidinone derivative	66.3	0.42
29	35.348	Dibutyl phthalate	89.7	0.39
30	36.249	Linoleic acid	71.5	0.95
31	38.069	Histidine derivative (methyl ester)	64.0	0.59
32	38.438	Diazahomoadamantane derivative	64.0	0.56

Note: GC–MS assignments were based on comparison with Wiley 7th Edition and NIST’02 mass spectral libraries. Authentic reference standards were not used; therefore, all compounds are reported as tentative or putative annotations rather than confirmed identifications. Entries with library match probabilities below 90% should be interpreted as putative annotations or chemical-class assignments.

**Table 8:** LC–QTOF–MS data and tentative annotations of polyphenolic compounds in the extract of *A. virgata*.

No.	Retention Time, min	Tentative Annotation	Molecular Formula	Molecular Weight [M], Da	Structural Formula
**1**	1.91	Scopoletin, 6-O-Methylesculetin	C_10_H_8_O_4_	192,168	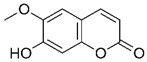
**2**	3.0	Dihydrocaffeic acid, 3-(3,4-Dihydroxyphenyl)-propanoic acid	C_9_H_10_O_4_	182,173	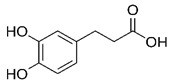
**3**	3.0	Nepetin;6-Methoxyluteolin	C_16_H_12_O_7_	316,262	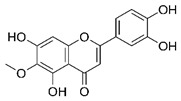
**4**	8.7	3-Caffeoylquinic acid	C_16_H_18_O_9_	354,309	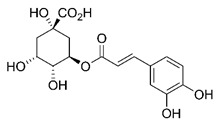
**5**	10.39	3-p-Coumaroylquinic acid	C_16_H_18_O_8_	338,309	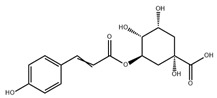
**6**	10.9	Quercetin 3,4′-O-diglucoside	C_27_H_30_O_17_	626,517	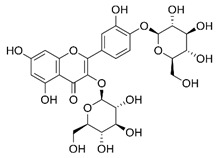
**7**	11.55	o-Coumaric acid	C_9_H_8_O_3_	164,158	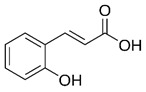
**8**	11.55	Luteolin 6-C-glucoside	C_21_H_20_O_11_	448,377	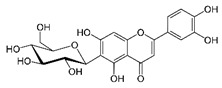
**9**	12.12	Quercetin 3-O-glucoside	C_21_H_20_O_12_	464,376	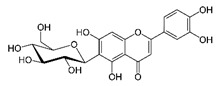
**10**	12.6	Daidzin 7-O-glucoside	C_21_H_20_O_9_	416,380	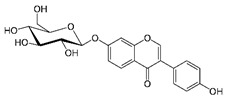
**11**	12.8	Quercetin 3-O-(6”-malonyl-glucoside)	C_24_H_22_O_15_	550,422	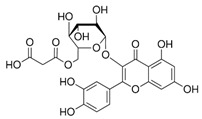
**12**	15.67	Caffeic acid ethyl ester	C_11_H_12_O_4_	208,211	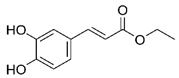
**13**	15.67	Quercetin	C_15_H_10_O_7_	302,236	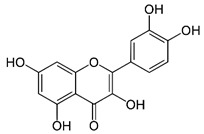
**14**	16.97	Apigenin	C_15_H_10_O_5_	270,237	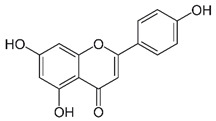
**15**	10.39	3,4-DHPEA-Elenolic acid glucoside	C_25_H_32_O_13_	540,514	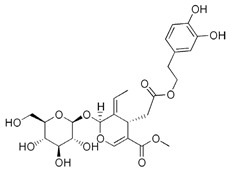
**16**	22.1	Caffeoyl tartaric acid	C_13_H_12_O_9_	312,229	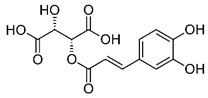
**17**	22.1	6″-O-Acetylglycitin	C_24_H_24_O_11_	488,441	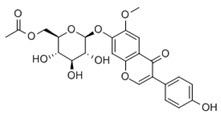
**18**	22.1	1-Sinapoyl-2-feruloylgentiobiose	C_33_H_40_O_18_	724,66	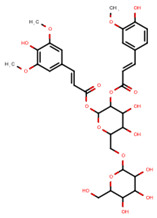
**19**	22.1	1,2,2′-Trisinapoylgentiobiose	C_45_H_52_O_23_	960,881	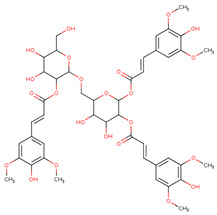

Note: LC–QTOF–MS assignments were based on accurate mass measurements, retention behavior, characteristic MS/MS fragment ions, and comparison with literature data and spectral databases. Authentic reference standards and NMR confirmation were not used; therefore, the compounds are reported as tentative annotations rather than confirmed identifications.

**Table 9:** LC–MS data and tentative annotations of polyphenolic compounds in the extract of *A. pyrifolia*.

No.	Retention Time (min)	Tentative Annotation	Molecular Formula	Molecular Weight [M, Da]	Structural Formula
**1**	1.9	Scopoletin (6-O-methylesculetin)	C_10_H_8_O_4_	192.168	* 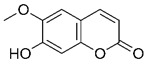 *
**2**	1.9	Oleuropein aglycone	C_19_H_22_O_8_	378.373	* 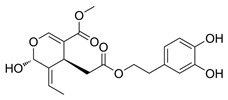 *
**3**	3.71	Gallic acid	C_7_H_6_O_5_	170	* 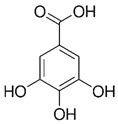 *
**4**	3.71	3,7-Dimethylquercetin	C_17_H_14_O_7_	330.289	* 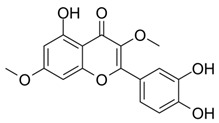 *
**5**	8.87	Dihydromyricetin 3-O-rhamnoside	C_21_H_22_O_12_	466.392	* 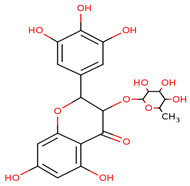 *
**6**	9.85	7-Oxomatairesinol	C_20_H_20_O_7_	372.369	* 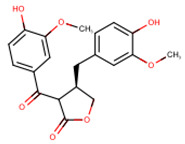 *
**7**	10.58	Catechin	C_15_H_14_O_6_	290.268	* 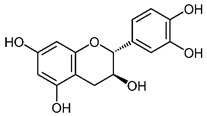 *
**8**	10.58	Myricetin 3-O-arabinoside	C_20_H_18_O_12_	450.350	* 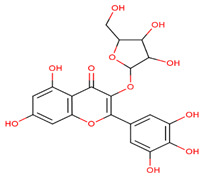 *
**9**	10.58	Quercetin 3-O-(6″-malonyl-glucoside)	C_24_H_22_O_15_	550.422	* 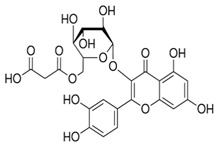 *
**10**	12.26	Trachelogenin	C_21_H_24_O_7_	388.411	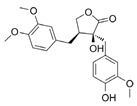
**11**	13.29	Luteolin 6-C-glucoside	C_21_H_20_O_11_	448.377	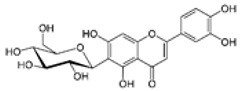
**12**	13.75	Quercetin 3-O-acetyl-rhamnoside	C_23_H_22_O_12_	490.414	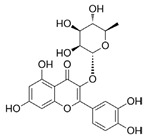
**13**	13.75	Kaempferol 3-O-(6″-malonyl-glucoside)	C_24_H_22_O_14_	534.423	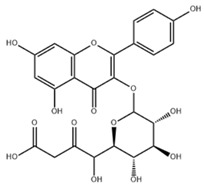
**14**	16.47	Dihydrocaffeic acid	C_9_H_10_O_4_	182.173	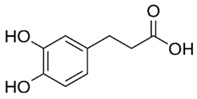
**15**	17.54	Luteolin	C_15_H_10_O_6_	286.236	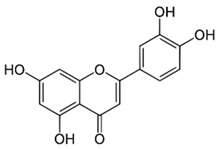
**16**	20.57	Procyanidin	C_30_H_26_O_12_	578.520	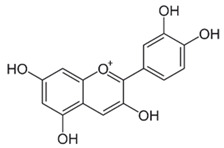
**17**	20.57	1,2,2′-Trisinapoylgentiobiose	C_45_H_52_O_23_	960	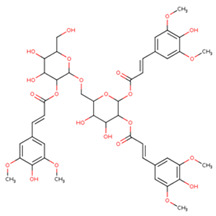

Note: LC–MS assignments were based on accurate mass measurements, retention behavior, characteristic MS/MS fragment ions, and comparison with literature data and spectral databases. Authentic reference standards and NMR confirmation were not used; therefore, the listed compounds should be interpreted as tentative annotations rather than confirmed identifications.

**Table 10:** Clinical Signs and Mortality of Mice after Single Oral Administration of *A. virgata* and *A. pyrifolia* Extracts at Different Doses.

Group (Dose)	Animal ID	Clinical Signs (Days of Observation)
		1–14 (All Days)
*A. virgata* (300 mg/kg)/*A. pyrifolia* (300 mg/kg)	1–1 to 1–5	Normal (γ)
*A. virgata* (500 mg/kg)/*A. pyrifolia* (500 mg/kg)	2–1 to 2–5	Normal (γ)
*A. virgata* (1000 mg/kg)/*A. pyrifolia* (1000 mg/kg)	3–1 to 3–5	Normal (γ)
*A. virgata* (2000 mg/kg)/*A. pyrifolia* (2000 mg/kg)	4–1 to 4–5	Normal (γ)

γ = normal (no clinical signs observed).

**Table 11:** Changes in body weight of mice after single oral administration of aqueous extracts of *A. virgata* and *A. pyrifolia* at different doses (mean ± SEM, n = 5, SEM = standard error of the mean).

Species	Group	Dose (mg/kg)	Before Experiment (g)	After 7 Days (g)	After 14 Days (g)
*A. virgata*	1	300	22.34 ± 0.26	25.26 ± 0.27	27.20 ± 0.21
	2	500	22.44 ± 0.30	25.06 ± 0.21	27.58 ± 0.62
	3	1000	22.68 ± 0.19	24.96 ± 0.51	27.36 ± 0.38
	4	2000	22.68 ± 0.42	25.56 ± 0.79	28.33 ± 0.31
*A. pyrifolia*	1	300	22.42 ± 0.42	25.46 ± 0.44	27.38 ± 0.50
	2	500	22.68 ± 0.59	25.76 ± 0.68	27.00 ± 0.79
	3	1000	22.84 ± 0.61	24.36 ± 0.60	27.71 ± 0.60
	4	2000	23.12 ± 0.46	26.66 ± 0.67	30.40 ± 1.02

**Table 12:** Absolute organ weights of mice after single oral administration of aqueous extracts of *A. virgata* and *A. pyrifolia* (mean ± SEM, n = 5, SEM = standard error of the mean).

Dose (mg/kg)	Liver (g)	Lungs (g)	Spleen (g)	Kidneys (g)	Heart (g)
*A. virgata*					
300	1.1542 ± 0.0268	0.2523 ± 0.0079	0.1024 ± 0.0021	0.4318 ± 0.0246	0.1468 ± 0.0050
500	1.6533 ± 0.0158	0.2592 ± 0.0103	0.1041 ± 0.0041	0.4898 ± 0.0249	0.1514 ± 0.0061
1000	1.1858 ± 0.1259	0.2587 ± 0.0136	0.1083 ± 0.0055	0.5321 ± 0.0071	0.1527 ± 0.0035
2000	1.4159 ± 0.1814	0.2623 ± 0.0038	0.0990 ± 0.0016	0.4358 ± 0.0146	0.1442 ± 0.0025
*A. pyrifolia*					
300	1.3555 ± 0.0806	0.2901 ± 0.0039	0.1036 ± 0.0047	0.2346 ± 0.0166	0.1411 ± 0.0064
500	1.5085 ± 0.0466	0.2480 ± 0.0187	0.1078 ± 0.0055	0.2748 ± 0.0166	0.1330 ± 0.0187
1000	1.5194 ± 0.0635	0.2432 ± 0.0225	0.0995 ± 0.0017	0.3621 ± 0.0269	0.1352 ± 0.0080
2000	1.6156 ± 0.1840	0.2391 ± 0.0258	0.1067 ± 0.0068	0.4696 ± 0.0636	0.1463 ± 0.0042

**Table 13:** Extraction parameters of *A. virgata* and *A. pyrifolia* under subcritical conditions.

Extracts	Extraction Parameters				Extract Yield (g, %)
	Extraction Mass (g)	Operating Pressure (kgf/cm^2^)	Extraction Temperature (°C)	Extraction Time (h)	
*A. virgata* (aerial parts)	670	57–65	18–23	8	5
*A. pyrifolia* (aerial parts)	660	57–65	18–23	8	10
*A. pyrifolia* (stems)	740	57–65	18–23	8	3

**Table 14:** Distribution of mice by groups for acute toxicity assessment of *A. virgata* and *A. pyrifolia* extracts following oral administration.

Test Substance	Group	Administration Volume (mL)	Dose (mg/kg)	Number of Animals
*A. virgata* extract	Experimental 1	0.5	300	5
*A. pyrifolia* extract	Experimental 2	0.5	500	5
	Experimental 3	0.5	1000	5
	Experimental 4	0.5	2000	5

## Data Availability

The original contributions presented in this study are included in the article and Supplementary Materials. Further inquiries can be directed to the corresponding author.

## Supplementary Materials

The following supporting information can be downloaded at https://www.mdpi.com/article/10.3390/molecules31111795/s1. Figure S1: Representative micrographs following administration of an aqueous extract of *Atraphaxis virgata*. Figure S2: Representative micrographs following administration of an aqueous extract of *Atraphaxis pyrifolia*.

## Abbreviations

GC–MS	Gas Chromatography–Mass Spectrometry
LC–MS	Liquid Chromatography–Mass Spectrometry
HPLC	High-Performance Liquid Chromatography
UHPLC	Ultra-High-Performance Liquid Chromatography
ESI	Electrospray Ionization
QTOF	Quadrupole Time-of-Flight
LC–ESI–QTOF–MS	Liquid Chromatography–Electrospray Ionization–Quadrupole Time-of-Flight–Mass Spectrometry
NMR	Nuclear Magnetic Resonance
SEM	Standard Error of the Mean
TIC	Total Ion Chromatogram
MS	Mass Spectrometry
LD_50_	Median Lethal Dose
OECD	Organisation for Economic Co-operation and Development
SD	Standard Deviation
NIST	National Institute of Standards and Technology (Mass Spectral Library)
SOP	Standard Operating Procedure
NOAEL	No-Observed-Adverse-Effect Level

## References

[1] Grudzinskaya L., Gemejiyeva N., Karzhaubekova Z., Nelina N. (2021). Botanical Coverage of the Leading Families of Medicinal Flora of Kazakhstan. BIO Web Conf..

[2] Pavlov N.V. (1966). Flora of Kazakhstan.

[3] Abdulina S.A., Kamelin R.V. (1998). List of Vascular Plants of Kazakhstan.

[4] Zhang Q., Yue Y., Li X., Zhang C., Guo Y., Wang Z., Li J. (2025). Advances in analytical techniques for bioactive compound quantification in medicinal plants: Innovations, challenges, and pharmaceutical applications. Microchem. J..

[5] Abilkassymova A., Kozykeyeva R., Aldana-Mejía J.A., Adams S.J., Datkhayev U., Turgumbayeva A., Orynbassarova K., Saroja S.G., Khan I.A., Ross S.A. (2024). Phytochemical and micro-morphological characterization of *Atraphaxis pyrifolia* Bunge growing in the Republic of Kazakhstan. Molecules.

[6] Yurtseva O.V., Deviatov A.G., Sokoloff D.D. (2022). Exocarp structure in the genus *Atraphaxis* (Polygonaceae, Polygoneae). Plant Syst. Evol..

[7] Yurtseva O.V., Kuznetsova O.I., Mavrodieva M.E., Mavrodiev E.V. (2016). What Is *Atraphaxis* L. (Polygonaceae, Polygoneae): Cryptic Taxa and Resolved Taxonomic Complexity Instead of the Formal Lumping and the Lack of Morphological Synapomorphies. PeerJ.

[8] Yurtseva O.V., Kuznetsova O.I., Mavrodiev E.V. (2016). A Broadly Sampled 3-Loci Plastid Phylogeny of *Atraphaxis* (Polygoneae, Polygonoideae, Polygonaceae) Reveals New Taxa: I. *Atraphaxis kamelinii* Spec. Nov. from Mongolia. Phytotaxa.

[9] Ibragimov M.K., Akmuradov A., Khodzhamberdyev Z.D. (2015). Some Endemic Medicinal Plants of Koytendaga in Gastroenterology. Exp. Clin. Gastroenterol..

[10] Khamreva D.T. (2023). New Findings in the Ethnobotany of Uzbekistan. Eurasia Proc. Health Environ. Life Sci..

[11] Odonbayar B., Murata T., Batkhuu J., Yasunaga K., Goto R., Sasaki K. (2016). Antioxidant Flavonols and Phenolic Compounds from *Atraphaxis frutescens* and Their Inhibitory Activities against Insect Phenoloxidase and Mushroom Tyrosinase. J. Nat. Prod..

[12] Wang X., Khutsishvili M., Fayvush G., Tamanyan K., Atha D., Borris R.P. (2018). Phytochemical Investigations of *Atraphaxis spinosa* L. (Polygonaceae). Biochem. Syst. Ecol..

[13] Frolova N., Bilova T., Silinskaia S., Orlova A., Gurina A., Frolov A. (2026). Gas Chromatography–Mass Spectrometry (GC-MS) in the Plant Metabolomics Toolbox: GC-MS in Multi-Platform Metabolomics and Integrated Multi-Omics Research. Int. J. Mol. Sci..

[14] Shin S., Kim S., Lee J., Son H., Paik J.-H., Gemejiyeva N.G., Karzhaubekova Z.Z., Lee T., Yoo H.Y. (2025). Investigation of *Atraphaxis virgata*, an Unexplored Medicinal Plant Rich in Flavonoids, as a Functional Material. Horticulturae.

[15] Kaufmann A. (2014). Combining UHPLC and high-resolution MS: A viable approach for the analysis of complex samples?. TrAC Trends Anal. Chem..

[16] Ali A., Bashmil Y.M., Cottrell J.J., Suleria H.A.R., Dunshea F.R. (2021). LC-MS/MS-QTOF Screening and Identification of Phenolic Compounds from Australian Grown Herbs and Their Antioxidant Potential. Antioxidants.

[17] Priya A.D., Martin A. (2025). UHPLC-MS/MS based comprehensive phenolic profiling, antimicrobial and antioxidant activities of Indian Rhodomyrtus tomentosa fruits. Sci. Rep..

[18] Murata T., Batkhuu J. (2021). Biological Activity Evaluations of Chemical Constituents Derived from Mongolian Medicinal Forage Plants and Their Applications in Combating Infectious Diseases and Addressing Health Problems in Humans and Livestock. J. Nat. Med..

[19] Panche A.N., Diwan A.D., Chandra S.R. (2016). Flavonoids: An overview. J. Nutr. Sci..

[20] Cory H., Passarelli S., Szeto J., Tamez M., Mattei J. (2018). The Role of Polyphenols in Human Health and Food Systems: A Mini-Review. Front. Nutr..

[21] Fraga C.G., Croft K.D., Kennedy D.O., Tomás-Barberán F.A. (2019). The effects of polyphenols and other bioactives on human health. Food Funct..

[22] Shahidi F., Yeo J. (2018). Bioactivities of Phenolics by Focusing on Suppression of Chronic Diseases: A Review. Int. J. Mol. Sci..

[23] Xie J., Xiong S., Li Y., Xia B., Li M., Zhang Z., Shi Z., Peng Q., Li C., Lin L. (2024). Phenolic acids from medicinal and edible homologous plants: A potential anti-inflammatory agent for inflammatory diseases. Front. Immunol..

[24] Chumbalov T.K., Mukhamed’yarova M.M., Omurkamzinova V.B., Chanysheva I.S. (1975). 7-O-Methylgossypetin 3-Rhamnoside from *Atraphaxis Pyrifolia*. Chem. Nat. Compd..

[25] Chumbalov T.K., Mukhamed’yarova M.M., Chanysheva I.S., Il’yasova M.M. (1971). Flavonoids of *Atraphaxis Pyrifolia* and Spinosa II. Chem. Nat. Compd..

[26] Chumbalov T.K., Mukhamed’yarova M.M., Chanysheva I.S., Smirnova L.P., Omurkamzinova V.B. (1976). Flavonoids of *Atraphaxis Pyrifolia*. III. Chem. Nat. Compd..

[27] Chumbalov T.K., Omurkamzinova V.B. (1976). Flavonoids of *Atraphaxis pyrifolia*. IV. Chem. Nat. Compd..

[28] Gupta P., Ahtisham, Nandi D., Ram S., Chhabra R., Bagal Y.S. (2024). Oxalate crystals and abiotic stress tolerance in plants. Plant Secondary Metabolites and Abiotic Stress.

[29] Liu S., Zheng J. (2024). Adaptive strategies based on shrub leaf–stem anatomy and their environmental interpretations in the eastern Qaidam Basin. BMC Plant Biol..

[30] Pan T., Britton T.G., Schrader J., Sumner E., Nicolle D., Choat B., Wright I.J. (2025). Adaptation in wood anatomical traits to temperature and precipitation—A common garden study. Plant Cell Environ..

[31] (2006). Carbon Dioxide, Gaseous and Liquid. Specifications.

[32] Dias A.L.B., de Aguiar A.C., Rostagno M.A. (2021). Extraction of natural products using supercritical fluids and pressurized liquids assisted by ultrasound: Current status and trends. Ultrason. Sonochem..

[33] Mohammadi M.A., Safavizadeh V., Yousefi M., Hosseini S.M. (2024). A short review of supercritical fluid extraction of plant extracts. J. Food Meas. Charact..

[34] Tzima S., Georgiopoulou I., Louli V., Magoulas K. (2023). Recent Advances in Supercritical CO_2_ Extraction of Pigments, Lipids and Bioactive Compounds from Microalgae. Molecules.

[35] Alseekh S., Aharoni A., Brotman Y., Contrepois K., D’Auria J., Ewald J., Ewald J.C., Fraser P.D., Giavalisco P., Hall R.D. (2021). Mass spectrometry-based metabolomics: A guide for annotation, quantification and best reporting practices. Nat. Methods.

[36] Da Porto C., Natolino A., Scalet M. (2022). Improved Sustainability in Wine Industry Byproducts: A Scale-up and Economical Feasibility Study for High-Value Compounds Extraction Using Modified SC-CO_2_. ACS Omega.

[37] Dauletova M.D., Umbetova A.K., Yelibayeva N.S., Burasheva G.S., Kabdraisova A.Z., Karzhaubekova Z.Z., Litvinenko Y.A., Assylkhanov Z.S., Korul’kin D.Y. (2025). Biologically Active Compounds of Plants of the *Atraphaxis* Genus: Chemical Composition and Immunomodulatory Evaluation. Int. J. Mol. Sci..

[38] López-Fernández O., Domínguez R., Pateiro M., Munekata P.E.S., Rocchetti G., Lorenzo J.M. (2020). Determination of Polyphenols Using Liquid Chromatography-Tandem Mass Spectrometry Technique (LC-MS/MS): A Review. Antioxidants.

[39] Waris M., Koçak E., Gonulalan E.M., Demirezer L.O., Kır S., Nemutlu E. (2022). Metabolomics analysis insight into medicinal plant science. TrAC Trends Anal. Chem..

[40] Valanciene E., Malys N. (2022). Advances in Production of Hydroxycinnamoyl-Quinic Acids: From Natural Sources to Biotechnology. Antioxidants.

[41] Gao X.Y., Li X.Y., Zhang C.Y., Bai C.Y. (2024). Scopoletin: A review of its pharmacology, pharmacokinetics, and toxicity. Front. Pharmacol..

[42] Neelam Khatkar A., Sharma K.K. (2020). Phenylpropanoids and its derivatives: Biological activities and its role in food, pharmaceutical and cosmetic industries. Crit. Rev. Food Sci. Nutr..

[43] Siniarska M., Bąbelewski P., Turkiewicz I.P., Wojdyło A. (2026). Profiling of polyphenolic compounds using LC–ESI–QTOF–MS/MS in fruits of the Sorbus subgenus as a valuable source of polyphenolic compounds. Food Chem..

[44] Ministry of Health of the Republic of Kazakhstan Order of the Acting Minister of Health of the Republic of Kazakhstan No. KR DSM-15 Dated 4 February 2021; Registered with the Ministry of Justice of the Republic of Kazakhstan on 9 February 2021, No. 22167. https://adilet.zan.kz/rus/docs/V2100022167?utm_source=chatgpt.com.

[45] OECD (2002). Test No. 423: Acute Oral Toxicity—Acute Toxic Class Method.

[46] Aguirre-García Y.L., Castillo-Manzanares A., Palomo-Ligas L., Ascacio-Valdés J.A., Campos-Múzquiz L.G., Esparza-González S.C., Rodríguez-Herrera R., Nery-Flores S.D. (2024). Toxicity Evaluation of a Polyphenolic Extract from *Flourensia cernua* DC through *Artemia* Lethality Assay, Hemolytic Activity, and Acute Oral Test. J. Toxicol..

[47] Tavares E.d.A., Guerra G.C.B., da Costa Melo N.M., Dantas-Medeiros R., da Silva E.C.S., Andrade A.W.L., de Souza Araújo D.F., da Silva V.C., Zanatta A.C., de Carvalho T.G. (2023). Toxicity and Anti-Inflammatory Activity of Phenolic-Rich Extract from *Nopalea cochenillifera* (Cactaceae): A Preclinical Study on the Prevention of Inflammatory Bowel Diseases. Plants.

[48] Sequeda-Castañeda L.G., Ospina-Giraldo L.F., Gutiérrez-Prieto S.J., Luengas-Caicedo P.E. (2025). Phytochemical Profile and Acute Toxicity in CD-1 Mice of the Hydroethanolic Extract and Butanolic Fraction of *Piper marginatum* Jacq. J. Xenobiotics.

[49] Abilkassymova A., Turgumbayeva A., Sarsenova L., Tastambek K., Altynbay N., Ziyaeva G., Blatov R., Altynbayeva G., Bekesheva K., Abdieva G. (2024). Exploring Four *Atraphaxis* Species: Traditional Medicinal Uses, Phytochemistry, and Pharmacological Activities. Molecules.

[50] Fettach S., Mrabti H.N., Sayah K., Bouyahya A., Salhi N., Cherrah Y., El Abbes F.M. (2019). Phenolic content, acute toxicity of *Ajuga iva* extracts and assessment of their antioxidant and carbohydrate digestive enzyme inhibitory effects. S. Afr. J. Bot..

[51] Unuofin J.O., Otunola G.A., Afolayan A.J. (2018). Evaluation of acute and subacute toxicity of whole-plant aqueous extract of *Vernonia mespilifolia* Less. in Wistar rats. J. Integr. Med..

[52] Wolff F.R., Broering M.F., Jurcevic J.D., Zermiani T., Bramorski A., Vitorino J.C., Malheiros A., Santin J.R. (2019). Safety assessment of *Piper cernuum* Vell. (Piperaceae) leaves extract: Acute, sub-acute toxicity and genotoxicity studies. J. Ethnopharmacol..

[53] Ministry of Health of the Republic of Kazakhstan (2009). State Pharmacopoeia of the Republic of Kazakhstan.

[54] Prozina M.N. (1960). Botanical Microtechnique.

[55] Dolgova A.A., Ladygina E.Y. (1977). Manual for Practical Training in Pharmacognosy.

[56] Yeung E.C.T., Stasolla C., Sumner M.J., Huang B.Q. (2015). Plant Microtechniques and Protocols.

[57] Joshi R., Faqeerzada M.A., Kim T., Cho B.-K. (2025). Recent advances in plant imaging technology: A concise review. Plant Image Sci..

[58] Yuan J., Wang X., Zhou H., Li Y., Zhang J., Yu S., Wang M., Hao M., Zhao Q., Liu L. (2020). Comparison of sample preparation techniques for inspection of leaf epidermises using light microscopy and scanning electron microscopy. Front. Plant Sci..

[59] Sozzani R., Busch W., Spalding E.P., Benfey P.N. (2014). Advanced imaging techniques for the study of plant growth and development. Trends Plant Sci..

[60] Barykina R.P., Veselova T.D., Devyatov A.G. (2004). Handbook of Botanical Microtechnique.

[61] Ministry of Health of the Republic of Kazakhstan (1987). State Pharmacopoeia of the USSR.

[62] Ministry of Health of the Republic of Kazakhstan (1990). State Pharmacopoeia of the USSR.

[63] Ministry of Health of the Republic of Kazakhstan (2008). State Pharmacopoeia of the Republic of Kazakhstan.

