# Supplementary material for: Phytochemical Profiling and Toxicological Evaluation of Atraphaxis virgata and Atraphaxis pyrifolia Extracts Using GC–MS and LC–MS
Source: Molecules. 2026 May 23;31(11):1795. doi: 10.3390/molecules31111795 (PMC13257668; doi:10.3390/molecules31111795)
Supplement: Supplementary file 1 [file molecules-31-01795-s001.zip › Figure S1.pdf]

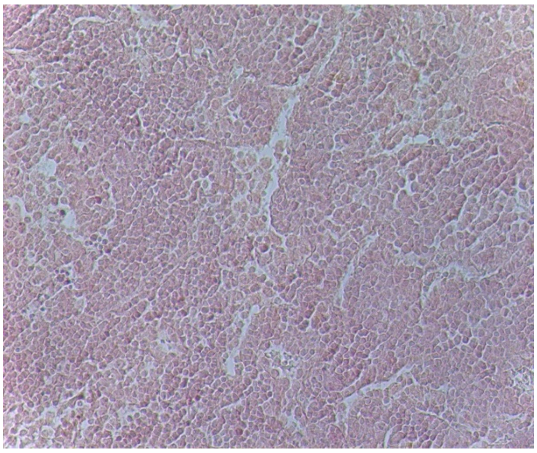

Spleen. Hematoxylin and eosin (H&E) staining.
Eyepiece ×10, objective ×20.
a

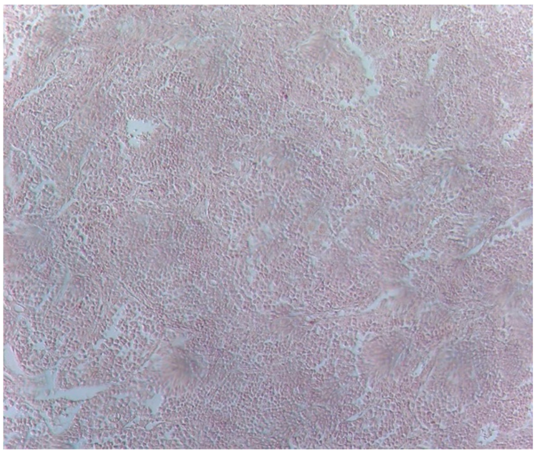

Spleen. Hematoxylin and eosin (H&E) staining.
Eyepiece ×10, objective ×10
b

Red pulp of the spleen. Diffuse edema.

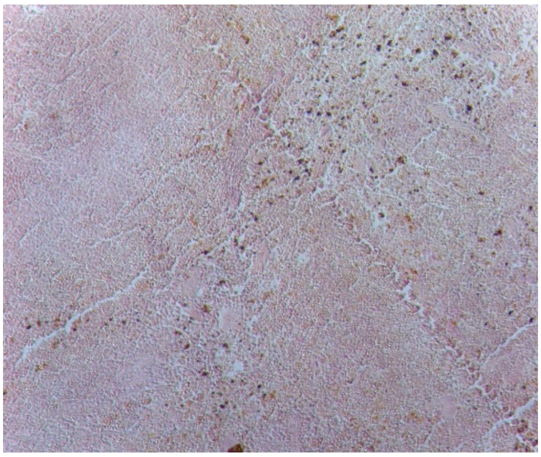

Spleen. Hematoxylin and eosin (H&E) staining.
Eyepiece ×10, objective ×20
c

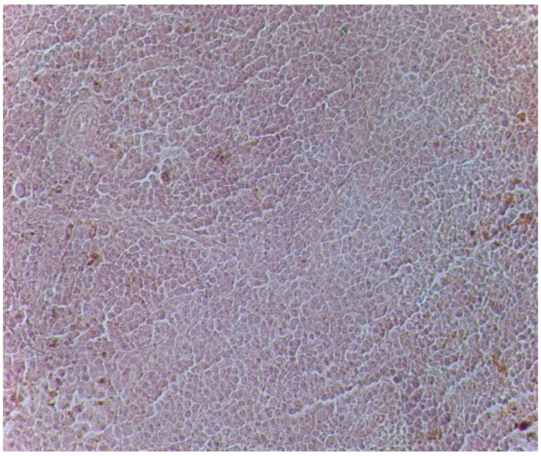

Spleen. Hematoxylin and eosin (H&E) staining.
Eyepiece ×10, objective ×10
d

Aggregates of siderophages are observed, and in some areas the pigment is associated with fibrous connective tissue replacing the normal elements of the red pulp (early stage of fibrosis).

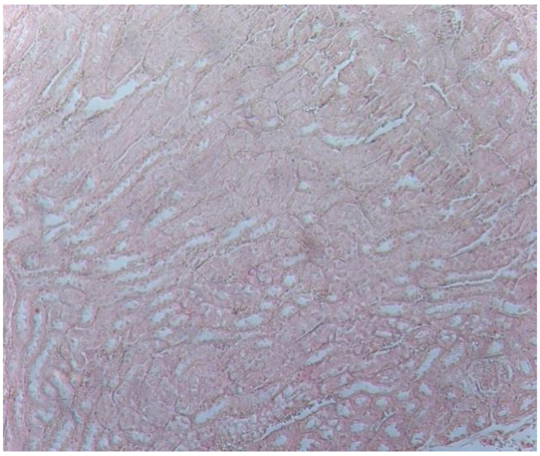

Kidney. Hematoxylin and eosin (H&E) staining.
Eyepiece ×10, objective ×10

e

Narrowing of the lumen of proximal and distal tubules at the corticomedullary junction of the kidney.

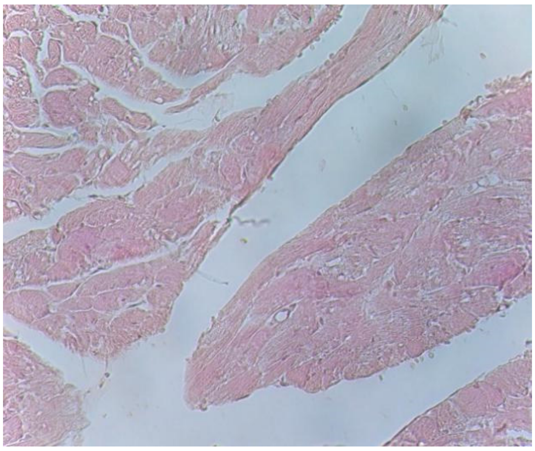

Heart. Hematoxylin and eosin (H&E) staining.
Eyepiece ×10, objective ×10

f

Dystrophic changes in cardiomyocytes; nuclei are displaced to the cell periphery or are indistinct

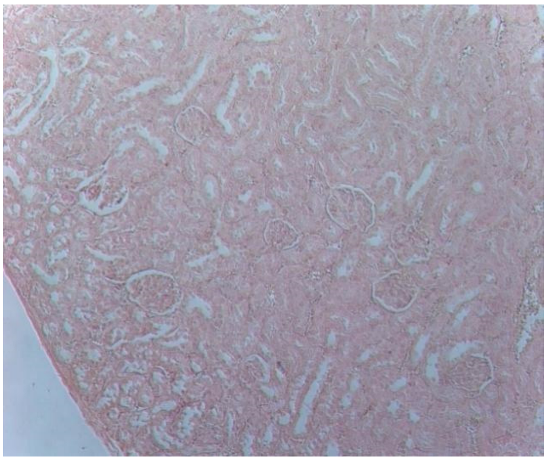

Kidney. Hematoxylin and eosin (H&E) staining.
Eyepiece ×10, objective ×10

g

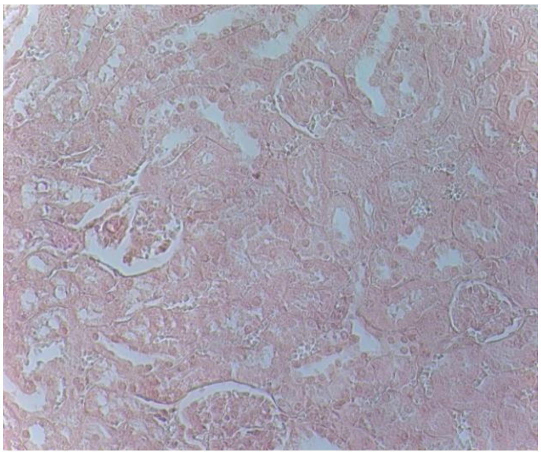

Kidney. Hematoxylin and eosin (H&E) staining.
Eyepiece ×10, objective ×20

h

Glomeruli with a clearly distinguishable urinary (Bowman's) space. In some cases, lobulation and mesangial hypercellularity are observed. Epithelial cells of the proximal tubules show nuclei displaced toward the lumen with a clarified perinuclear zone.

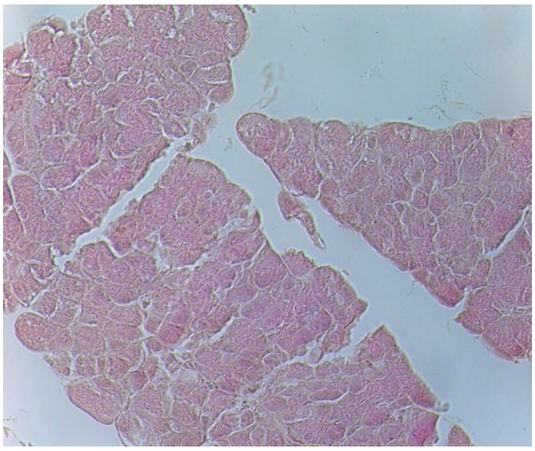

Pancreas. Hematoxylin and eosin (H&E) staining.
Eyepiece ×10, objective ×20
i

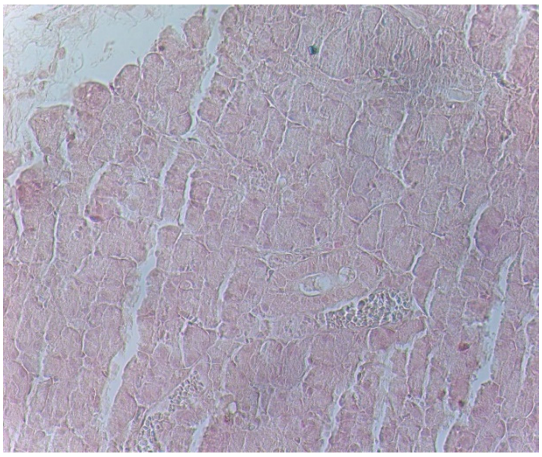

Pancreas. Hematoxylin and eosin (H&E) staining.
Eyepiece ×10, objective ×20
j

The acinar glands consist of basophilic cells with fine granularity.

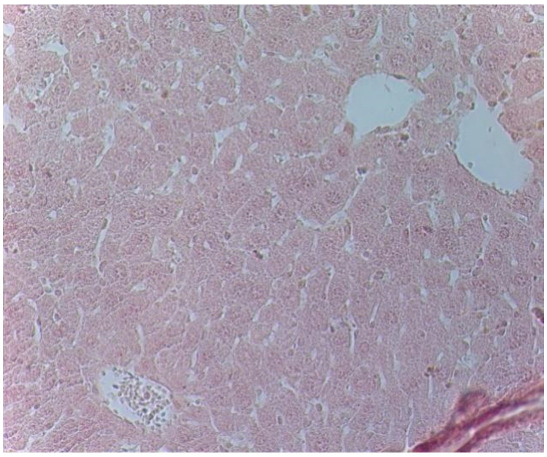

Liver. Hematoxylin and eosin (H&E) staining.
Eyepiece ×10, objective ×20
k

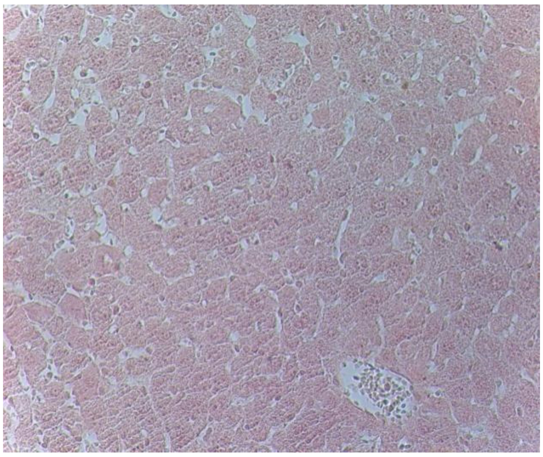

Liver. Hematoxylin and eosin (H&E) staining.
Eyepiece ×10, objective ×20
l

Hepatocytes exhibit two types of nuclear arrangement: large centrally located nuclei and smaller nuclei with a clear perinuclear zone. Diffuse dilation of the Disse spaces and activation of stellate (Ito) cells are observed.

Figure 9. Representative micrographs following administration of an aqueous extract of *Atraphaxis virgata*.
