# Supplementary material for: Phytochemical Profiling and Toxicological Evaluation of Atraphaxis virgata and Atraphaxis pyrifolia Extracts Using GC–MS and LC–MS
Source: Molecules. 2026 May 23;31(11):1795. doi: 10.3390/molecules31111795 (PMC13257668; doi:10.3390/molecules31111795)
Supplement: Supplementary file 1 [file molecules-31-01795-s001.zip › Figure S2.pdf]

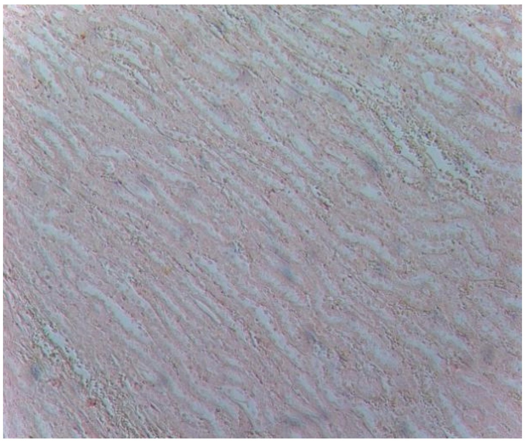

Kidney. Hematoxylin and eosin (H&E) staining. Eyepiece ×10, objective ×10

a

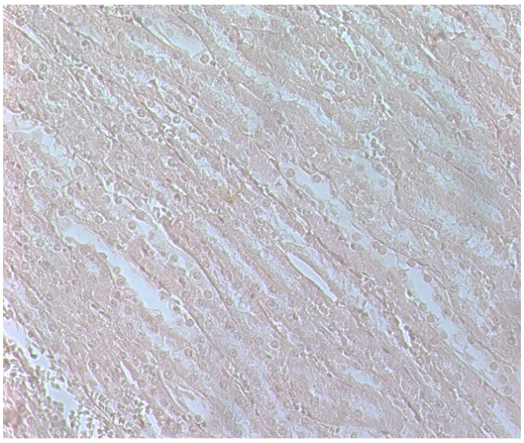

Kidney. Hematoxylin and eosin (H&E) staining. Eyepiece ×10, objective ×20

b

Medulla. Epithelial cells of all tubular types show nuclei displaced toward the lumen. In cells preserved on the basement membrane, a clear perinuclear space is observed.

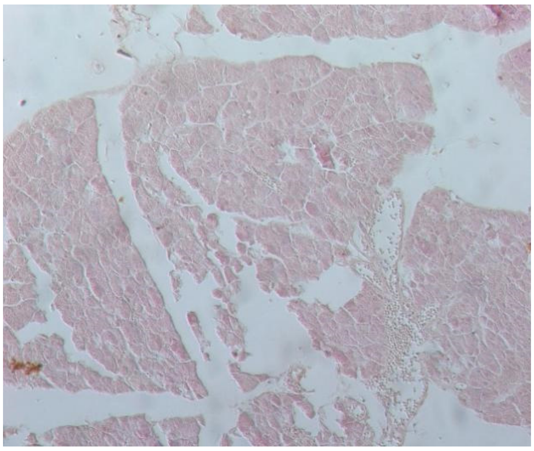

Pancreas. Hematoxylin and eosin (H&E) staining. Eyepiece ×10, objective ×20

c

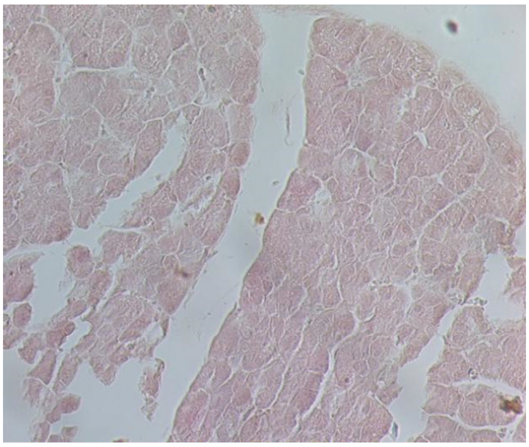

Pancreas. Hematoxylin and eosin (H&E) staining. Eyepiece ×10, objective ×40

d

Lobules of the acinar glands show no luminal dilation and contain secretory granules. The acinar glands consist of basophilic cells with fine granularity. No activation or increase in islet cells is observed. Focal lymphocytic infiltration and edema are present.

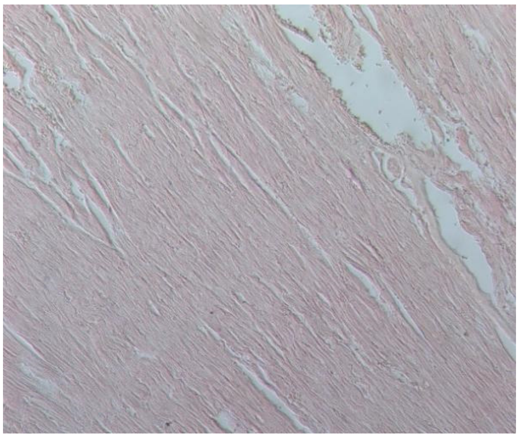

Heart. Hematoxylin and eosin (H&E) staining.
Eyepiece ×10, objective ×20
e

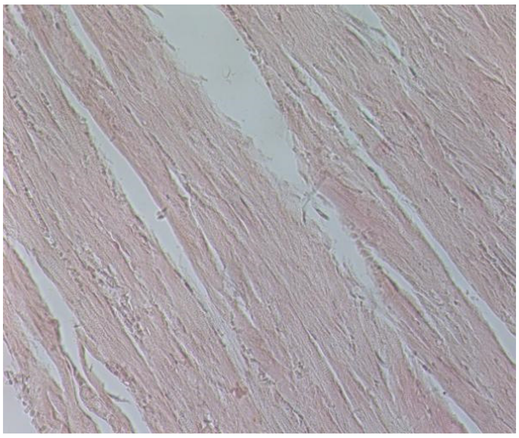

Heart. Hematoxylin and eosin (H&E) staining.
Eyepiece ×10, objective ×20
f

Cross-striation of cardiomyocytes is weakly expressed.

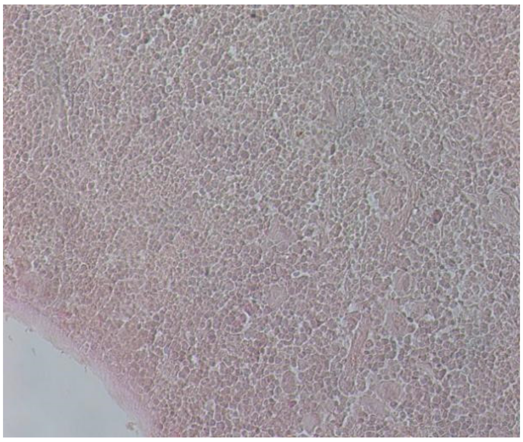

Spleen. Hematoxylin and eosin (H&E) staining.
Eyepiece ×10, objective ×20
g

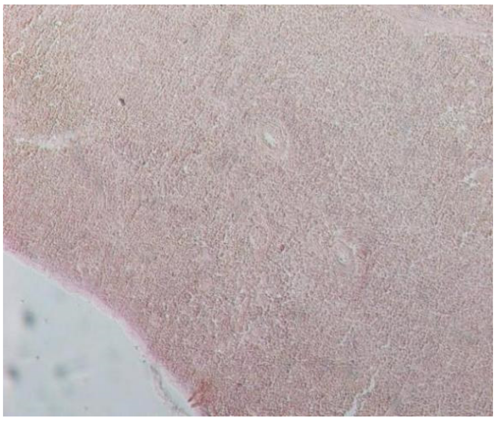

Spleen. Hematoxylin and eosin (H&E) staining.
Eyepiece ×10, objective ×10
h

Evidence of macrophage-mediated erythrophagocytosis. Plasma cells are identifiable.

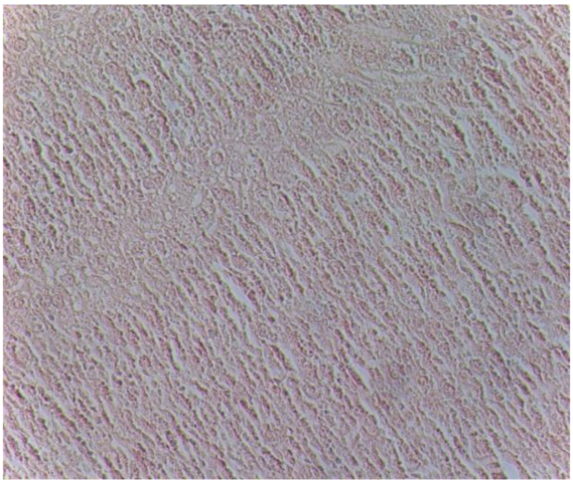

Hematoxylin and eosin (H&E) staining.
Eyepiece ×10, objective ×20
i

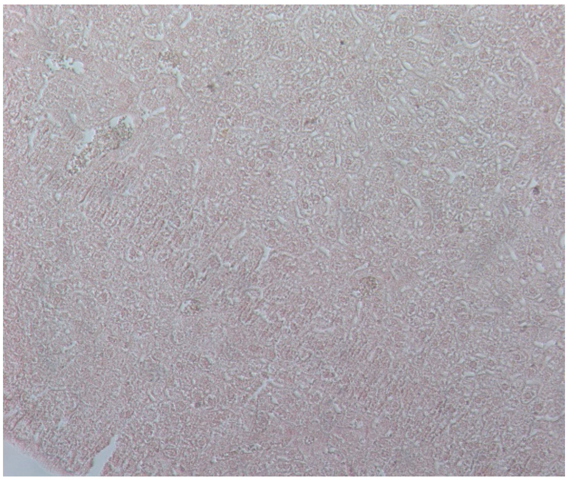

Liver. Hematoxylin and eosin (H&E) staining.
Eyepiece ×10, objective ×10
j

Necrobiosis of hepatocytes. Preserved hepatocytes show large centrally located nuclei with a pronounced clear perinuclear zone. Hepatocytes exhibit microvesicular and focal to moderate macrovesicular degeneration.

Figure 10. Representative micrographs following administration of an aqueous extract of *Atraphaxis pyrifolia*.
